# Genomic signals of migration and continuity in Britain before the Anglo-Saxons

**DOI:** 10.1038/ncomms10326

**Published:** 2016-01-19

**Authors:** Rui Martiniano, Anwen Caffell, Malin Holst, Kurt Hunter-Mann, Janet Montgomery, Gundula Müldner, Russell L. McLaughlin, Matthew D. Teasdale, Wouter van Rheenen, Jan H. Veldink, Leonard H. van den Berg, Orla Hardiman, Maureen Carroll, Steve Roskams, John Oxley, Colleen Morgan, Mark G. Thomas, Ian Barnes, Christine McDonnell, Matthew J. Collins, Daniel G. Bradley

**Affiliations:** 1 Smurfit Institute of Genetics, School of Genetics and Microbiology, Trinity College Dublin, Dublin 2, Ireland; 2 York Osteoarchaeology Ltd, 75 Main Street, Bishop Wilton, York YO42 1SR, UK; 3 Department of Archaeology, Dawson Building, Durham University, South Road, Durham DH1 3LE, UK; 4 BioArCh, Biology, S Block, Wentworth Way, York YO10 5DD, UK; 5 York Archaeological Trust for Excavation and Research Limited, 47 Aldwark, York YO1 7BX, UK; 6 Department of Archaeology, University of Reading, Whiteknights PO Box 227, Reading RG6 6AB, UK; 7 Department of Neurology and Neurosurgery, Brain Center Rudolf Magnus, University Medical Center Utrecht, Heidelberglaan 100, 3584 CX Utrecht, The Netherlands; 8 Academic Unit of Neurology, Trinity Biomedical Sciences Institute, Trinity College Dublin, Pearse Street, Dublin 2, Ireland; 9 Department of Archaeology, University of Sheffield Northgate House, West Street, Sheffield S1 4ET, UK; 10 City of York Council, York YO1 6GA, UK; 11 Research Department of Genetics, Evolution and Environment, University College London, Gower Street, London WC1E 6BT, UK; 12 Department of Earth Sciences, Natural History Museum, Cromwell Road, London SW7 5BD, UK

## Abstract

The purported migrations that have formed the peoples of Britain have been the focus of generations of scholarly controversy. However, this has not benefited from direct analyses of ancient genomes. Here we report nine ancient genomes (∼1 ×) of individuals from northern Britain: seven from a Roman era York cemetery, bookended by earlier Iron-Age and later Anglo-Saxon burials. Six of the Roman genomes show affinity with modern British Celtic populations, particularly Welsh, but significantly diverge from populations from Yorkshire and other eastern English samples. They also show similarity with the earlier Iron-Age genome, suggesting population continuity, but differ from the later Anglo-Saxon genome. This pattern concords with profound impact of migrations in the Anglo-Saxon period. Strikingly, one Roman skeleton shows a clear signal of exogenous origin, with affinities pointing towards the Middle East, confirming the cosmopolitan character of the Empire, even at its northernmost fringes.

## Abstract (web summary)

Here, Martiniano *et al*. examine the genetic structure of northern Britain in the late BC/early AD using ancient genome sequencing of 9 individuals. They uncover evidence of far-reaching Roman and later Anglo-Saxon migrations within a background of Britons similar to the earlier Iron Age.

## Graphical abstract

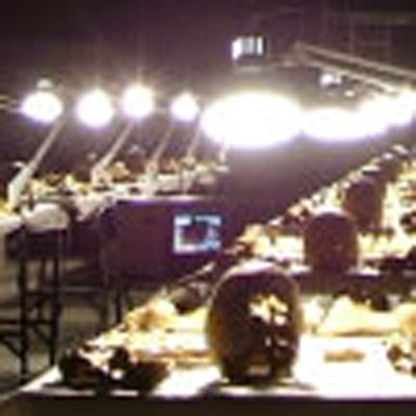

Ancient genomics has the power to anchor the emergence of modern genetic patterns to archaeological events but, to date, no such genome-scale data have emerged for the Romano-British world, or indeed from any era in the British past. Extensive surveying of modern genomic variation in the British Isles has produced divergent interpretations of the migratory history of the islands. An east–west gradient of Y chromosome, autosomal and mtDNA allele frequencies has been interpreted as reflecting the genetic legacy of substantial Anglo-Saxon invasions following the Roman period^1^,^2^,^3^,^4^. However, it is difficult to distinguish the effects of this much-debated event from other migratory influences from northwest continental Europe, whether these are, for example, Germanic elements in the Late Roman army predating the Anglo-Saxon migrations or Scandinavian settlers arriving some centuries afterwards^5^.

At its maximum, the Roman Empire stretched from Atlantic Europe to the Near East and from Northern Britain to the Sahara, incorporating an advanced transport infrastructure, which would have enabled previously unprecedented levels of mobility^6^. Evidence for the presence of foreigners in Britain has been based on epigraphic sources, material culture and, more recently, bioarchaeological (isotopic) data^7^,^8^,^9^. However, there is no way of knowing how representative the people mentioned in inscriptions might be; artefactual imports indicate contact beyond the province but may not point to movement of people; and isotopic baseline values in British data overlap considerably with numerous other regions, including much of Western Europe and the Mediterranean littoral^10^. Thus, measuring who moved about the Roman Empire, and on what scale, remains challenging.

To investigate the genetic identity of Britain in late BC and the early centuries AD we report shotgun genomic sequencing of nine human genomes to coverage depth of ∼1 × and analysis of these in the context of extensive genome-wide data from modern populations. Seven ancient genomes are sampled from a cemetery in Roman York dated between the second and the fourth century AD, one from an earlier Yorkshire Iron-Age burial (210 BC–40 AD) and one from a later neighbouring Anglo-Saxon burial (650–910 AD).

## Results

### Archaeological samples

York (*Eboracum*), founded c. AD 71, became the Roman empire's northernmost provincial capital in about AD 200. Its southwest approach road was lined with tombstones and mausolea^11^ and excavations conducted there between 2004 and 2005 at Driffield Terrace revealed a cemetery dating from the second to the fourth century AD with a high incidence of decapitated remains^12^. After initial screening of eight individuals chosen for DNA extraction, seven presented superior endogenous DNA content. Near Melton, East Yorkshire, a first century AD late Iron-Age settlement gave human remains predominantly of adult females and non-adults^13^ from which five individuals were sampled. From these, the best preserved individual with known date was M1489 (between 210 BC and 40 AD). Norton Bishopsmill, dates to 650–910 AD and was a Christian Anglo-Saxon cemetery excavated in the village of Norton, Teesside, northeast England^14^. We sampled 3 individuals from burials of 100 skeletons and selected the best preserved, NO3423, for the present study (Supplementary Notes 1 and 2 and Supplementary Fig. 1).

### Sequencing results and sample contamination

Gamba *et al.*^15^ established that the dense internal centre of the inner ear petrous bone is an excellent source of preserved ancient DNA suitable for high-throughput sequencing. Accordingly, we sampled single petrous bones, extracted DNA and made indexed Illumina sequencing libraries. After preliminary screening for endogenous content, nine samples were chosen for genome-level sequencing. On alignment to the human reference genome, reads showed mismatch patterns typical of archaeological DNA indicating deamination damage (Supplementary Fig. 6). Contamination estimated from both mtDNA heterozygosity (1.82±0.47%) and X-chromosome contamination in male samples (0.79±0.21%) was low (Supplementary Note 2 and Supplementary Tables 5–7).

### Sex determination and uniparental marker analysis

Using the ratio between sequencing reads aligned to the X and Y chromosomes^16^, it was possible to assign biological sex to each individual, confirming skeletal assessments: the Anglo-Saxon and each Roman-period sample were male, whereas the Iron-Age sample was female (Supplementary Fig. 7). Mitochondrial genomes were retrieved for each sample with between 39 × and 98 × coverage and were assigned to known haplogroups (Table 1), which are common in present-day European populations^17^. Y-chromosome haplogroups were determined for each male (Table 1): the majority (6/7) of Driffield Terrace samples belong to sub-lineages of R1b-L52/L11, which reaches its highest frequencies (>70%) in Western European countries^18^. Sample 3DRIF-26, on the other hand, despite belonging to the same burial context, presented a lineage consistent with haplogroup J2-L228, which has a modern distribution centred on the Middle East, but which is also present in the Caucasus region, the Balkans and Italy^19^. The Anglo-Saxon (NO3423) sample was assigned to haplogroup I1-S107, which is widespread in Nordic countries^20^.

### Affinity with global populations

**Figure 1:**
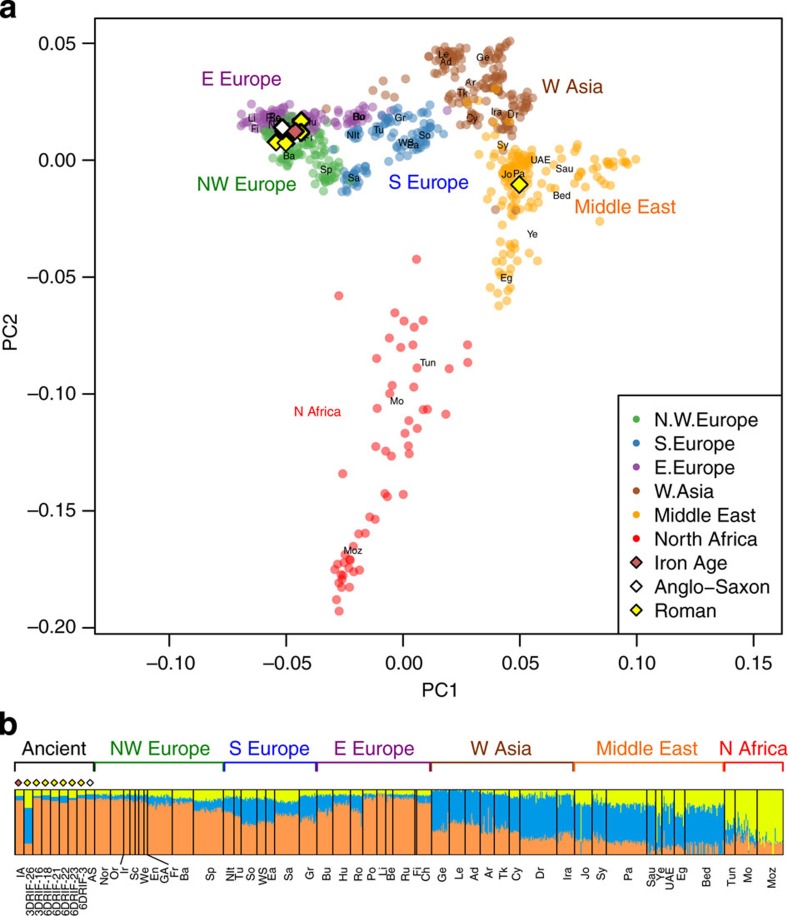
Principal component analysis. (**a**) PCA and (**b**) model-based clustering using NGSadmix (*K*=3) of Driffield Terrace, Iron-Age and Anglo-Saxon samples merged with European, West Asian, Middle Eastern and North African populations^21^. Population key: Ad, Adygei; Ar, Armenian; Ba, Basque; Bed, Bedouin; Be, Belorussian; Bu, Bulgarian; Ch, Chuvash; Cy, Cypriot; Dr, Druze; Ea, East Sicilian; Eg, Egyptian; En, English; Fi, Finnish; Fr, French; Ge, Georgian; GA, Germany/Austria; Gr, Greek; Hu, Hungarian; Ira, Iranian; Ir, Ireland; Jo, Jordanian; Le, Lezgin; Li, Lithuanian; Mo, Moroccan; Moz, Mozabite; Nit, North Italian; Nor, Norwegian; Or, Orcadian; Pa, Palestinian; Po, Polish; Ro, Romanian; Ru, Russian; Sa, Sardinian; Sau, Saudi; Sc, Scottish; So, South Italian; Sp, Spanish; Sy, Syrian; Tun, Tunisian; Tk, Turkish; Tu, Tuscan; UAE, United Arab Emirates; We, Welsh; WS, West Sicilian; Ye, Yemeni.

We called between ∼210 and ∼400 thousand single-nucleotide polymorphisms (SNPs) within our ancient samples that had previously been genotyped in a data set of 780 European, West Asian, North African and Middle Eastern individuals^21^. Figure 1a shows a principal component analysis (PCA) where eight of nine ancient genomes cluster close to a collection of northwest European samples. One York Roman 3DRIF-26 gives a clear Middle Eastern signal, with closest neighbours of Palestinian, Jordanian and Syrian origin. This dichotomy is also apparent in maximum likelihood estimation of individual ancestries using NGSadmix (Fig. 1b). In this, when a model of three ancestral populations is imposed across the entire sample, this analysis highlights three major geographical foci: Europe; North Africa; and West Asia/Middle East. The European ancestral component predominates in the majority of ancient samples (which show similar profiles to modern northwestern Europeans), whereas 3DRIF-26 again shows a majority West Asian/Middle Eastern component. Isotopic analyses of the skeletons support this genetic differentiation of 3DRIF-26 from the remainder of the individuals sampled. Strontium isotope ratios (^87^Sr/^86^Sr) vary mainly according to geological substrate, while oxygen isotope values (∂^18^O), which track locally available drinking water, reflect climatic and geographic variables such as temperature, rainfall levels or distance from the coast^22^. When we compared these ratios in our seven samples with other British Romans, 3DRIF-26 showed both an unusually low ^87^Sr/^86^Sr ratio and an extreme ∂^18^O_p_ value (Supplementary Fig. 2).

**Figure 2:**
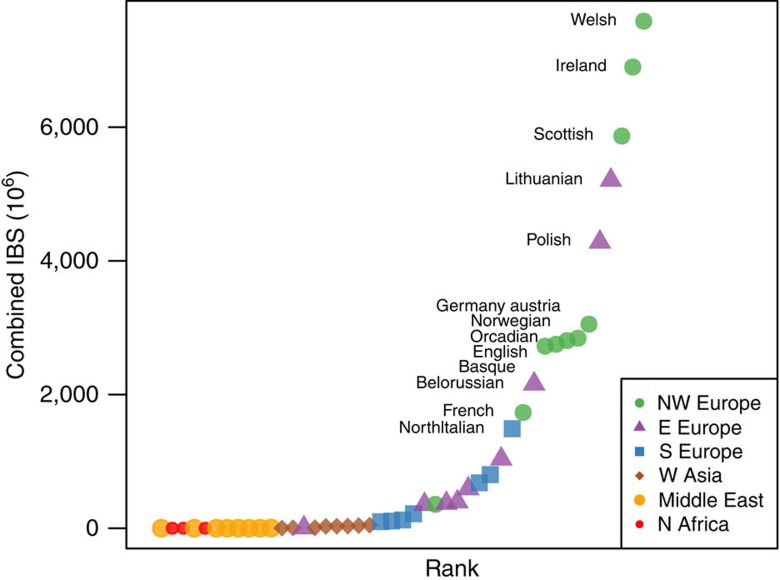
Combined percentile scores of modern European samples ranked by IBS to the Roman York genotypes. IBS reveals strongest affinity to modern Welsh, followed by Irish and Scottish. One outlier 3DRIF-26 was excluded from this analysis.

To maximize resolution of genetic affinity, for each ancient sample we performed pairwise comparisons with each modern sample and calculated the proportion of SNP positions at which these were identical by state (IBS). Only single-SNP alleles were considered at each locus and were randomly sampled from the biallelic genotype. Taking the median IBS score for each modern population sample, we then ranked these for similarity to each ancient genome in turn. Interestingly, the top-ranked modern sample for IBS for each of these ancient British samples was one of the formerly Celtic language-speaking regions of the British Isles, with the single exception of 3DRIF-26, which showed highest IBS with samples from Saudi Arabia. We gauged the sensitivity of this approach by checking whether individual modern samples were assignable to their region of origin. When tested, local individuals were assigned with high frequency (0.97) to the British Isles and also most often to their correct country. The method showed lower sensitivity for Middle Eastern genotypes, with primary assignment to that region in only 39% of instances (Supplementary Fig. 15). Nevertheless, outside assignments tended to be to Cypriot, Sardinian and Druze, never to Northern Europe. In contrast, specificity of a Middle Eastern assignment was high—only three individuals, from Iran, Tunisia and Morocco, were incorrectly assigned to that region. Thus, assignment of 3DRIF-26 to the Middle East region seems secure, but resolution to an individual population may not be possible. Specificity in assignment to the British Isles was lower, with about half of assignments (0.53) derived from elsewhere, most often from neighbouring populations such as France (0.28) and Norway (0.15). Small sample sizes (∼10 per population) render individual scores only weakly informative but, when we compared the six Roman burials after excluding the outlying 3DRIF-26, their rank orders across the geographical sample were highly correlated (Spearman rank correlation coefficients (*r*=0.982; *P*<0.01; Supplementary Note 2, Supplementary Figs 12–14 and Supplementary Tables 11 and 12). This allowed us to consider these together and generate a combined percentile score by scoring each comparator population as the product of the rank percentiles versus each Roman genotype (Fig. 2). The Welsh were most consistently ranked as highly identical by state, followed by Irish and Scottish scores, a result strongly supporting an origin within the British Isles for this Roman sample majority. Interestingly, the modern English sample was ranked only ninth for IBS to the Romans from York, at a level similar to German, Norwegian, Orcadian and Basque samples.

### Ancient sample ancestry within Britain

**Figure 3:**
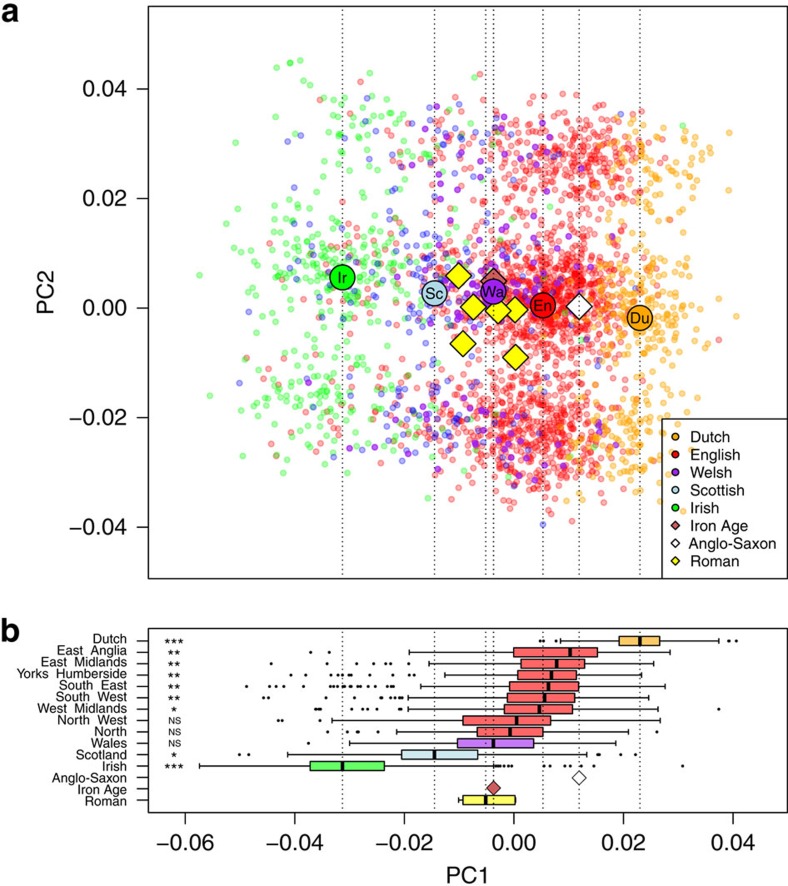
Principal component analysis. (**a**) PCA of the Roman samples from Driffield Terrace (excluding one outlier), one Iron-Age individual and one Anglo-Saxon merged with modern Irish, British and Dutch genotype data. (**b**) Boxplot of PC1 broken down by subregion. The symbols on the left represent the significance of a Mann–Whitney test performed to compare the Roman population with all other populations in the data set. There were no significant differences between the Roman sample and the present-day Welsh, Northern and North Western English samples included in this analysis; all other regions had significantly different median values for PC1. Population key: Du, Dutch; En, English; Ir, Irish; NS, not significant; Sc, Scottish; Wa, Wales. NS-*P*>0.05; *0.05>*P*>0.01; **0.01>*P*>0.0001; ***P<0.0001.

To place our ancient genomes within a detailed British context, we next plotted these in a background PCA using 3,075 published genotypes from British^3^, Irish^23^ and southern Netherlands samples^24^. The modern samples were analysed using SNP genotypes at ∼250,000 loci and projected into a single plot using smartpca (Fig. 3a). As in Burton *et al.*^3^ the first component of the variation was informative for the structure within Britain. Given the close ancestral relationships between these populations and their well-known history of migrational exchange, a substantial overlap between regional groups was both expected and observed. However, by considering median values, one can see a clear progression from Irish samples at one pole through Scottish, Welsh, English to the Dutch cohort at the other extreme. In this plot the York Romans cluster centrally close to the modern Welsh median value, along with the Iron-Age genome. The local Anglo-Saxon is placed differently, closest to modern East Anglians between the English and Dutch medians.

This first component also offers an opportunity to compare within the English sample. Figure 3b shows a boxplot of PC1 values for each subsample and structure is evident, with higher median values in Eastern regions such as East Anglia, East Midlands, intermediate values in the southern and western parts and lower values in the north and northwest. This pattern is more clearly seen in a geographical plot of interpolated values (Fig. 5a). When the York Romans are compared together with each modern cohort, they are most similar to the Welsh distribution of PC1 values and differ significantly from all other regional groups, apart from those from North and Northwest England (Mann–Whitney test; Fig. 3b, Supplementary Note 2 and Supplementary Table 13). An interesting difference is the marked one between the Driffield Terrace ancient and contemporary Yorkshire samples (*P*=0.003), implying regional discontinuity. It is also worth noting that the PC1 coordinate of the Anglo-Saxon individual is closer to the median PC1 value of East Anglians, possibly reflecting a more pronounced contribution of Germanic immigrants to eastern British populations. However, we note the inherent uncertainty in drawing inference from a single sample.

**Figure 4:**
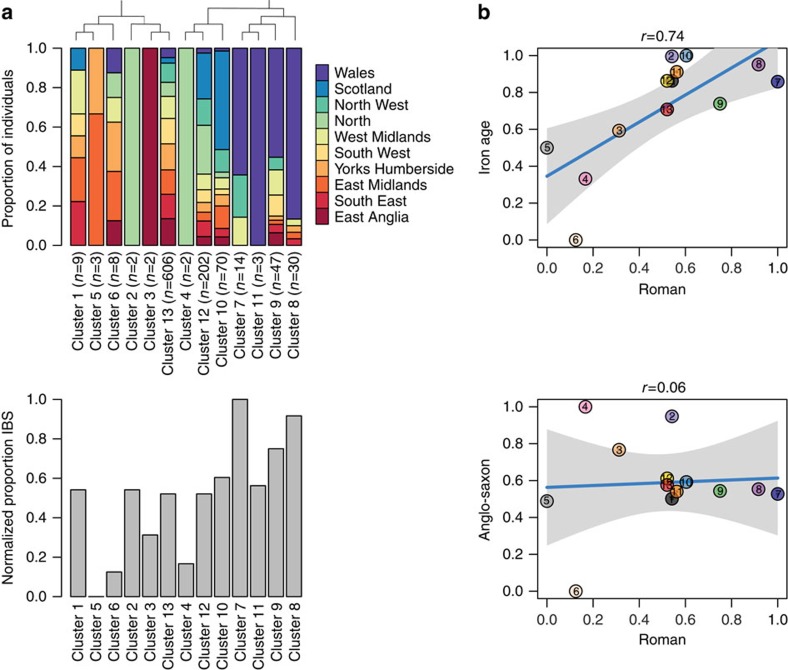
FineSTRUCTURE analysis of modern British genotypes and IBS affinity to the British Roman cohort. (**a**) The inferred clusters of moderns, their regional origins, the order of emergence of these groups and numbers of individuals in each.Below, median IBS between each cluster and the ancient Roman samples is plotted; the most prominent feature is their relative similarity to the predominantly Welsh clusters. (**b**) Plots of median cluster IBS values of the Romans versus the single Iron-Age genome and, below, versus the Anglo-Saxon sample. The strong relationship in the former is some indication of Iron-Age Roman genetic continuity, whereas discontinuity between Romans and the Anglo-Saxon is supported by their lack of correlation.

Leslie *et al.*^4^ used a haplotype-based clustering method, implemented in fineSTRUCTURE^25^ to deconstruct British populations into geographically and historically meaningful clusters. Accordingly, we used this approach to search for substructure within the modern British genotypes used here and to identify patterns of allele sharing between the ancient samples and the clusters identified. Structure was apparent with separation into subgroups of predominantly Welsh, English and Scottish provenance. The six Romans shared most alleles on average with those clusters consisting primarily of Welsh individuals (Fig. 4 and Supplementary Fig. 16). Allele sharing patterns also allowed comparison with the other ancients; when median IBS values across the clusters were compared between those for the Iron-Age genome and those for the Roman cohort, these correlated strongly (*r*=0.74, *P*=0.004), supporting continuity. However, a comparison between the Anglo-Saxon and the Romans showed no correlation (*r*=0.06, *P*=0.842; Fig. 4).

**Figure 5:**
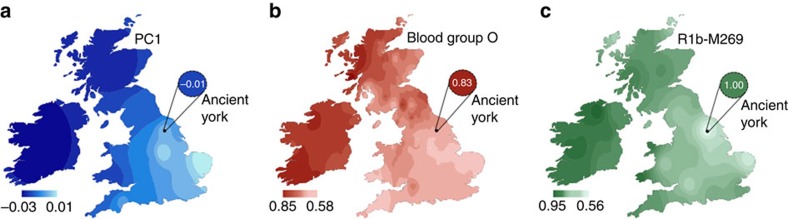
Interpolated maps of allele frequency comparing Roman York samples and modern populations from the British Isles. (**a**) PC1 median values; (**b**) blood group O frequency; (**c**) Y-chromosome haplogroup R1b1a2-M269 frequency.

Genomic change in Yorkshire between the early centuries AD and modern sampling is further illustrated by both the oldest and most extensively typed genetic system (the ABO blood group) and the system known to show maximal differentiation within the British Isles (chromosome Y haplogroup R1b1a2-M269). The plots in Fig. 5 show imputed blood group O and observed chromosome Y haplogroup R1b1a2-M269 frequencies for the Roman genomes (excluding the immigrant outlier) contrasting sharply with interpolated allele frequencies for modern eastern Britain^1^,^26^,^27^,^28^.

### Imputation and phenotype determination

Using a similar approach to that of Gamba *et al.*^15^, we used phased reference genomes from the 1000 Genomes Project to impute genotypes associated with phenotypic traits. In particular, we inferred genotypes at SNP positions to predict eye and hair pigmentation^29^. The most common predicted phenotype in the Roman burial samples is brown eyes and black/brown hair. However, one sample, 6DRIF-18, was estimated to have had a distinctive appearance with blue eyes and blonde hair, as did the single Anglo-Saxon individual. We also inferred that blood group O is the most common in the Roman samples (Supplementary Table 17). The Iron-Age sample is also estimated as blood type O and the Anglo-Saxon is likely to have been type B or possibly type A. Five samples returned imputed lactase persistence genotypes: two Roman burials and the Iron-Age individual were likely to have been lactase persistent, while two Romans, 6DRIF-22 and the suspected migrant 3DRIF-26 were homozygous for the ancestral non-persistence variant.

## Discussion

Combined genomic and isotopic evidence support the inference that the origins and childhood of individual 3DRIF-26 lay far outside Britain. His modern genomic affinities clearly lie with the Middle East. Isotopically, the most plausible suggestion is an arid environment on igneous or limestone geology, which is consistent with the same regions (Supplementary Fig. 2 and Supplementary Note 2). Hence, although this individual is indistinguishable from the other inhumations in terms of burial practice and osteology, the analyses show that, even in its northernmost provincial capital, the profoundly cosmopolitan nature of the Roman Empire suggested by documentary and epigraphic sources continued to hold sway.

The peoples of Britain show marked genetic structure (Fig. 5), which has been the focus of generations of investigation^30^. A common theme in this research has been a contrast between a southern and eastern lowland zone and a western and northern upland zone. This patterning resembles the geography of Anglo-Saxon settlement in the fifth to seventh centuries AD, inviting the conclusion that the cultural and linguistic change effected by this migration was also reflected by major genetic change^1^,^2^,^31^.

Projections from modern data to the past are, however, subject to considerable uncertainties and may be compounded by unknown complexities, which do not feature in their underlying models. Prehistorians point out that the Germanic affinity of eastern Britain could also be a result of earlier communications with the northwest European mainland. For example, there may have been ‘Belgic' peoples in Britain at the time of the Claudian conquest in AD43 (ref. 32), and the Roman army that arrived in Britain was composed of recruits from various provinces^33^. Recently Leslie *et al.*^4^ have used haplotype-based statistical methods applied to modern genome-wide SNP genotypes to infer several distinct ancestral influences from migratory events into Britain. This included a major 35% contribution to modern Central and Southern English populations from a German source, which, they surmise, occurred in the century after AD 800, some 200 years or more after archaeological evidence for initial Anglo-Saxon influence. Evidence from direct observations of ancient genomes is required, however, if we are to draw conclusions about genetic exchange that distinguish between closely dated events.

Six of the seven individuals sampled here are clearly indigenous Britons in their genomic signal. When considered together, they are similar to the earlier Iron-Age sample, whilst the modern group with which they show closest affinity are Welsh. These six are also fixed for the Y-chromosome haplotype R1b-L51, which shows a cline in modern Britain, again with maximal frequencies among western populations. Interestingly, these people differ significantly from modern inhabitants of the same region (Yorkshire and Humberside) suggesting major genetic change in Eastern Britain within the last millennium and a half. That this could have been, in some part, due to population influx associated with the Anglo-Saxon migrations is suggested by the different genetic signal of the later Anglo-Saxon genome. Iron-Age, Roman, Anglo-Saxon, Viking and other migrations have all been proposed as contributors to the genetic structure in modern United Kingdom^34^.

The thesis that the mountainous regions of Wales may have held populations that are representative of earlier, more widely dispersed indigenous British genetic strata is not new, yet it finds some support in our analyses. The genomes of modern Scottish and Irish populations diverge from this group of early inhabitants of northern Britain, whereas their Welsh counterparts do not. Modern data for genetic structure among non-Saxon samples from the British Isles are said to deny the existence of a single ‘Celtic' population^4^. Our data indicate that differentiation within such groups may have happened before the early centuries AD. By the same token, it lends support for genetic exchange between Scotland and Ireland, as attested in some historical sources and mirrored by linguistic affinity: Irish and Scottish Gaelic are sister P-Celtic languages, whereas Welsh is a divergent Q-Celtic language, akin to that thought to have been spoken throughout pre-Roman Britain^35^.

In the Roman York burials at Driffield Terrace, the majority were adults under 45 years old, male and most had evidence of decapitation^36^. They were slightly taller than average for Roman Britain, displayed a high occurrence of trauma, potentially related to interpersonal violence and evidenced childhood stress and infection (Supplementary Tables 1 and 2 and Supplementary Note 1). This demographic profile resembles the population structure in a recently excavated burial ground of the second and third century AD at Ephesus, which has been interpreted to be a burial ground for gladiators^37^. However, the evidence could also fit with a military context; the Roman army had a minimum height for recruitment^38^ and fallen soldiers would match the young adult profile of the cemetery. In this later Roman period increasingly large numbers of soldiers were enlisted locally^33^.

Whichever the identity of the enigmatic headless Romans from York, our sample of the genomes of seven of them, when combined with isotopic evidence, indicate six to be of British origin and one to have origins in the Middle East. This is the first refined genomic evidence for far-reaching ancient mobility and (although from an unusual context) also the first snapshot of British genomes in the early centuries AD, indicating continuity with an Iron-Age sample before the migrations of the Anglo-Saxon period.

## Methods

### Isotope analysis

Partial isotope data for six of the seven Driffield Terrace individuals have been previously published^7^,^39^. We sampled a molar tooth from the remaining individual 3DRIF-26 for isotope analysis and re-attempted collagen extraction on long bones of individuals where rib samples had previously failed to yield a viable product. Analyses were done at the NERC Isotope Geosciences Laboratory, Keyworth and Stable Isotope Laboratories, Universities of Bradford and Reading (UK). (Supplementary Note 2, Supplementary Figs 2–5 and Supplementary Tables 3 and 4).

### DNA extraction and sequencing

Ancient DNA sample processing was done at the Ancient DNA lab, Smurfit Institute, Trinity College Dublin (Ireland), in clean-room facilities exclusively dedicated to this purpose. We extracted DNA from ∼150 mg of nine temporal bone samples belonging to the Iron-Age (1), Roman (7) and Anglo-Saxon (1) burial sites in York (UK; Supplementary Note 1) using a modified^40^ silica-column-based method^41^. DNA libraries were constructed from extracted DNA using the method described in ref. 42 with modifications^15^,^43^. We amplified the aDNA (ancient DNA) libraries with 3–4 distinct indexing oligos for each sample to increase index diversity in each lane. PCR products were then purified (Qiagen MinElute PCR Purification Kit, Qiagen, Hilden, Germany), quantified (Agilent Bioanalyzer 2100) and pooled. Each sample was sequenced to approximately 1 × in an Illumina HiSeq 2000 (100 cycle kit, single-end reads mode; Macrogen; Supplementary Note 2).

### Read processing and analysis

Next-generation sequencing reads were trimmed with Cutadapt v. 1.3 (ref. 44). Two bases from each side of reads were removed with seqtk (https://github.com/lh3/seqtk). Reads were aligned to the human reference genome (UCSC hg19) and mtDNA (rCRS, NC_012920.1) with Burrows-Wheeler Aligner v.0.7.5a-r405 (ref. 45), filtering by base quality 15, discarding PCR duplicates and reads with mapping quality inferior to 30 using SAMtools v.0.1.19-44428cd (ref. 46; Table 1). Base quality scores were rescaled with mapDamage v.2.0 (ref. 47) to exclude potential deamination residues from subsequent analysis.

### Contamination estimates and authenticity

To determine the extent of contamination in the ancient samples sequenced, we calculated the number of mismatches in mtDNA haplotype defining mutations^15^ and X-chromosome polymorphisms in samples determined to be male (Supplementary Note 2 and Supplementary Tables 5–8)^48^. We also used PMDtools^49^ to select reads with evidence of deamination and compared sex determination and PCA (Supplementary Note 2 and Supplementary Figs 7 and 8). Finally, we confirmed the presence of aDNA misincorporations by analysing a subset of 1 million reads for each sample with mapDamage 2.0 (ref. 47).

### Sex determination and uniparental lineage determination

We used the method published in ref. 16 to determine the sex of the ancient individuals (Supplementary Fig. 7). Y-chromosome lineages of ancient male samples were identified using clean_tree software^50^ (http://www.erasmusmc.nl/fmb/resources/cleantree/; Supplementary Table 10). Regarding mtDNA analysis, reads were separately aligned to the revised Cambridge Reference Sequence (rCRS; NC_012920.1)^51^, filtering for base (*q*≥20) and mapping (*q*≥30) quality and duplicate reads as above. We then used SAMtools to obtain a consensus sequence in fasta format, which we uploaded to HaploFind^52^, which determines mtDNA haplogroups based on Phylotree build 16 (ref. 53) (Table 1 and Supplementary Note 2). Mitochondrial mutations detected in each sample are shown in Supplementary Table 9.

### Population genetics analysis and data sets

Alleles identified with Genome Analysis Toolkit v.2.5 (ref. 54), Pileup mode, were haploidized following ref. 55. For comparisons with modern human populations, we used two data sets: 780 individuals of European, Middle Eastern, West Asian and North African populations from ref. 21 and the other of Wellcome Trust Case Control Consortium (WTCCC1) 1958 British Birth Cohort SNP genotype data^3^ with Dutch^24^ and Irish^23^ genotypes (Supplementary Note 2). PCA (Figs 1a and 3a and Supplementary Figs 8 and 9) were performed using smartpca from the EIGENSOFT v.5 package^56^ and LASER 2.02 (ref. 57; Supplementary Fig. 10). Model-based clustering analysis was done with ADMIXTURE v.1.23 (ref. 58). We then extracted genotype likelihoods from aDNA data with ANGSD v.0.592 (ref. 48; Supplementary Fig. 11), which we combined with genotype data of present-day populations. We analysed this data with NGSadmix v.32 (ref. 59; *K*=3; Fig. 1b and Supplementary Note 2).

### Identity-by-state analysis

IBS between ancient and present-day samples was estimated with PLINK v.1.9 (ref. 60). Median IBS proportions between aDNA samples and European, Middle Eastern and North African populations were obtained and plotted individually on maps (Supplementary Fig. 12). Then, we selected only the Roman York samples, except for 3DRIF-26 (Middle Eastern affinity), ranked their median IBS score in relation to modern populations and combined these ranks by calculating their product (Fig. 2). Spearman rank correlations were estimated with R (ref. 61; Supplementary Note 2 and Supplementary Table 11). Regarding the WTCCC1 genotypes, we followed the same approach as described above, but present scaled median IBS values to the 0–1 range on Fig. 4.

### fineSTRUCTURE analysis

We randomly selected 100 individuals from each region of the WTCCC1 data set, excluding SNPs with missing genotypes, which resulted in a total of 431,366 variants and 1,000 samples. We used SHAPEIT2 to phase genotypes^62^ and ran the ChromoPainter pipeline^25^ with default parameters as implemented by fineSTRUCTURE v.2. For the fineSTRUCTURE analysis, the following settings were used: 3,000,000 burn in iterations, 1,000,000 sample iterations for the Markov chain Monte Carlo (MCMC) and 10,000,000 tree comparisons (Supplementary Note 2). We then called genotypes in our ancient samples for estimation of IBS between these and the inferred fineSTRUCTURE population clusters (Fig. 4 and Supplementary Figs 16 and 17).

### Imputation of phenotype associated loci

To investigate loci associated with selective sweeps, we took a similar approach as in ref. 15, where alleles observed in the 1000 Genomes Project^63^ were called with Genome Analysis Toolkit v.2.5, from which we extracted genotype likelihoods and converted to BEAGLE format. BEAGLE v.3.3.2 (ref. 64) was used to phase and subsequently impute genotypes at SNP positions described in the HIrisplex system^29^, loci associated with blood groups^65^,^66^, lactase persistence^67^,^68^ and pigmentation phenotypes^69^,^70^. Only posterior genotype probabilities ≥0.85 were kept^15^ (Supplementary Note 2 and Supplementary Table 14–17). We generated interpolated frequency maps of blood group^26^ and Y-chromosome frequency data^1^,^27^,^28^ with ArcMap v.10.1 from the ArcGis suite (Environmental Systems Research Institute) using the default settings of the geospatial analysis plugin (Fig. 4).

## Additional information

**Accession codes:** Raw Illumina sequencing reads can be downloaded at http://www.ebi.ac.uk/ena/data/view/PRJEB11004.

**How to cite this article:** Martiniano, R. *et al.* Genomic signals of migration and continuity in Britain before the Anglo-Saxons. *Nat. Commun.* 7:10326 doi: 10.1038/ncomms10326 (2016).

## Supplementary Material

Supplementary Information supplementary Figures 1-17, Supplementary Tables 1-18, Supplementary Note 1-2 and Supplementary References

## Figures and Tables

**Table 1:** Result summary for the samples analysed in the present study.

**Sample**	**Period**	**Excavation site**	**Total reads**	Mapped reads*	**Duplication %**	Endogenous %*	**Mean coverage (X)**	**chrY hg**	**mtDNA hg**
3DRIF-16	Roman	Driffield Terrace	63,341,920	28,683,678	2.92	43.96	0.67	R1b1a2a1a1-M405	H6a1a
3DRIF-26	Roman	Driffield Terrace	207,248,970	50,652,260	8.2	22.44	1.13	J2-L228	H5
6DRIF-18	Roman	Driffield Terrace	98,083,358	41,157,853	3.78	40.38	1.07	R1b1a2a1a-L52/L11	H1bs
6DRIF-21	Roman	Driffield Terrace	91,887,701	48,712,821	5.19	50.26	1.16	R1b1a2a1a2c2-DF63	J1c3e2
6DRIF-22	Roman	Driffield Terrace	115,324,680	45,995,965	2.45	38.91	1.12	R1b1a2a1a2b-S28	H2+195
6DRIF-23	Roman	Driffield Terrace	117,230,764	25,256,982	2.85	20.93	0.65	R1b1a2a1a-L52	H6a1b2
6DRIF-3	Roman	Driffield Terrace	112,316,793	68,421,310	2.59	59.34	1.67	R1b1a2a1a1-M405	J1b1a1
M1489	Iron Age	Melton	81,838,435	21,802,991	2.00	26.64	0.56	-	U2e1e
NO3423	Anglo-Saxon	Norton on Tees	89,918,177	43,369,123	2.00	48.23	1.05	I1-S107	H1a

^*^Reads were filtered by base quality 15, mapping quality 30 and duplicates removed.
