# Supplementary material for: Genomic signals of migration and continuity in Britain before the Anglo-Saxons
Source: Nat Commun. 2016 Jan 19;7:10326. doi: 10.1038/ncomms10326 (PMC4735653; doi:10.1038/ncomms10326)
Supplement: Supplementary Information — supplementary Figures 1-17, Supplementary Tables 1-18, Supplementary Note 1-2 and Supplementary References [file ncomms10326-s1.pdf]

Supplementary Figures

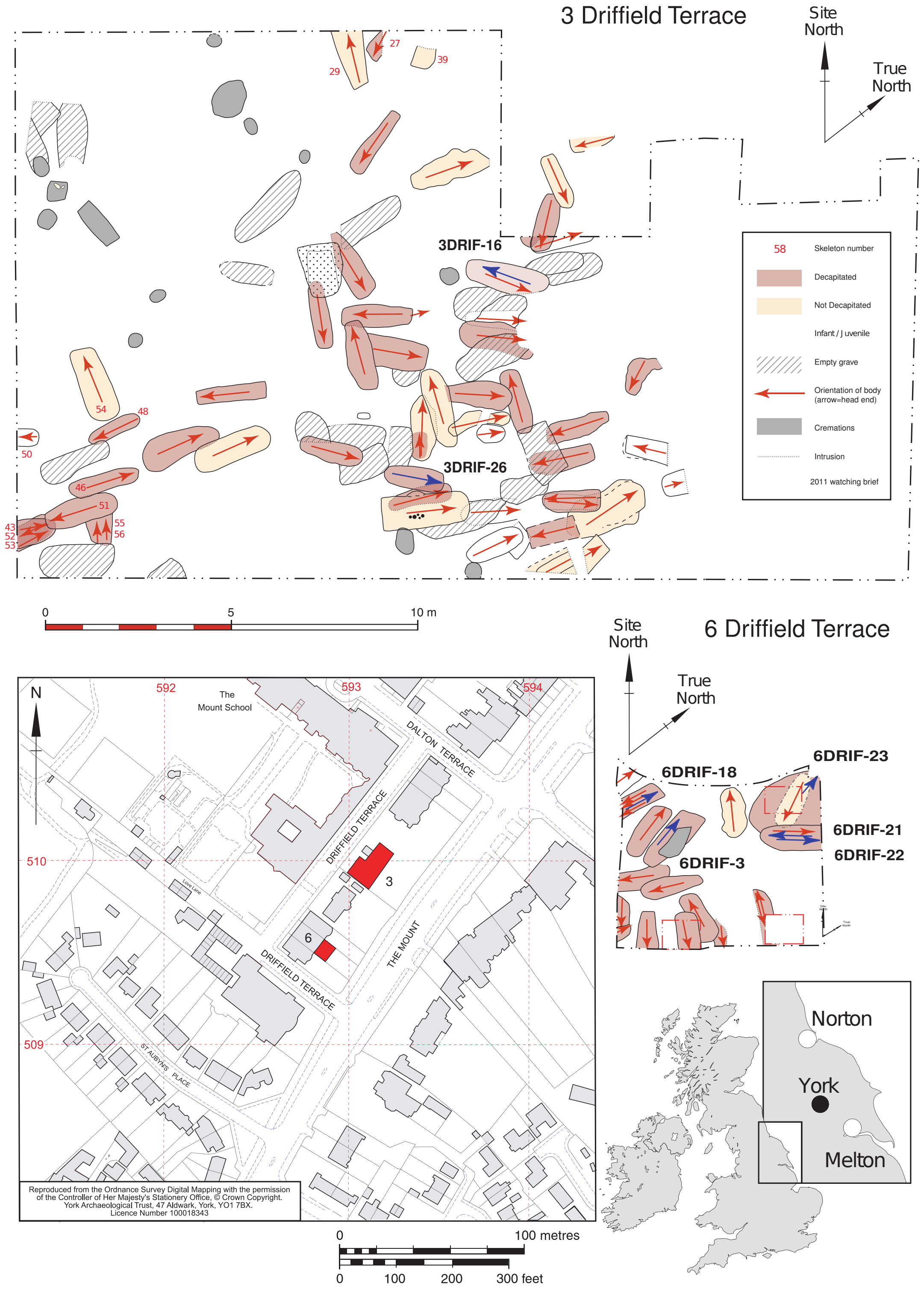

Supplementary Figure 1 - Location of Driffield Terrace, Norton on Tees and Melton Samples.

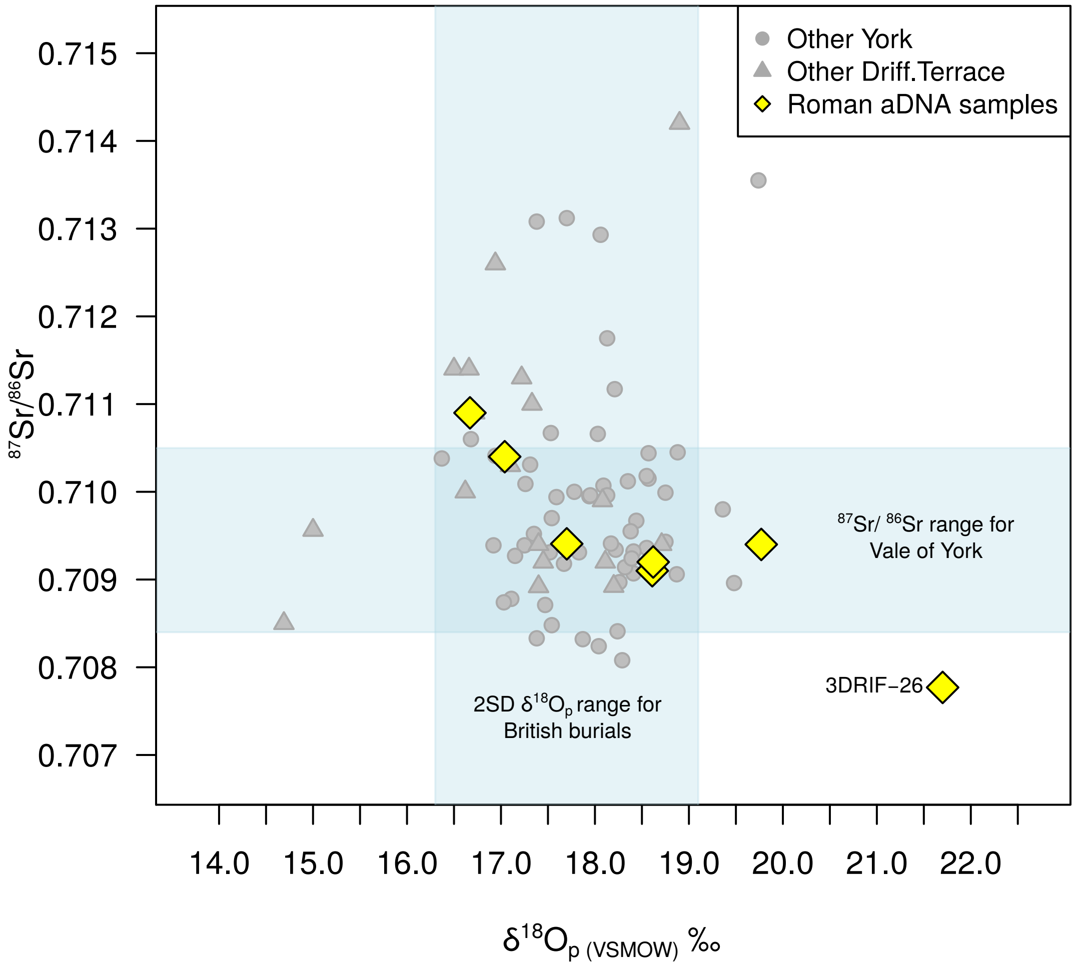

Supplementary Figure 2 - δ18O vs 87Sr/86Sr in tooth enamel carbonate from Driffield Terrace with samples selected for DNA highlighted and compared with other samples from Roman York. Current best estimates for the local 87Sr/86Sr and the British phosphate oxygen isotope (δ18Op) range are also indicated.

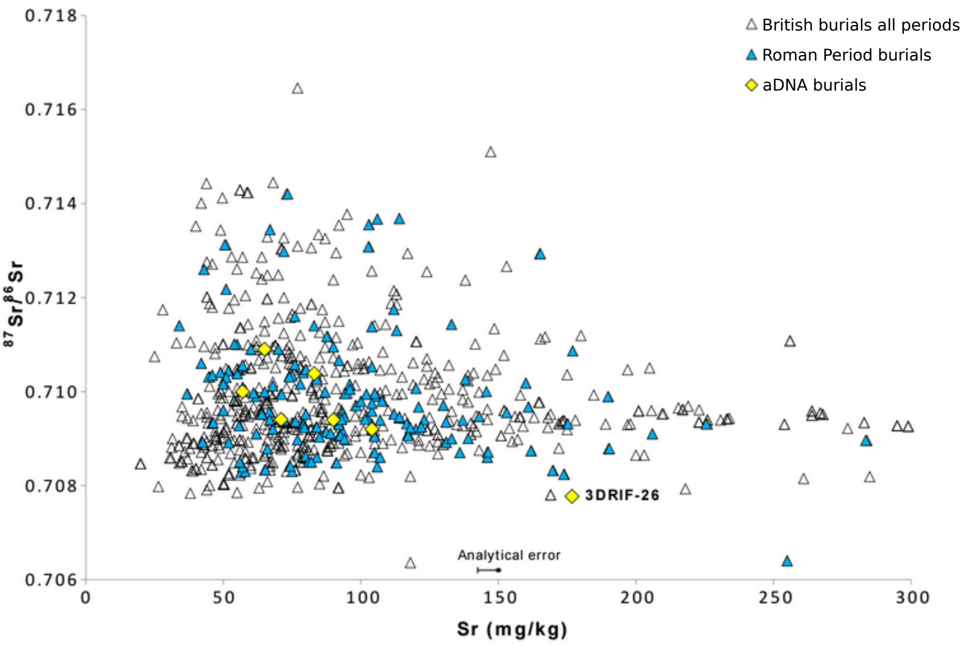

Supplementary Figure 3 - A biplot showing the distribution of strontium isotope and concentration data for archaeological human enamel excavated in Britain for: all periods; the Roman Period; and the seven individuals from Driffield Terrace, York analysed for aDNA. As the data are drawn from multiple sources 1–6 analytical uncertainty is estimated at +/- 0.002 % (2 SD) for 87Sr/86Sr (i.e. within symbol) and +/- 10 mg/kg for strontium concentration.

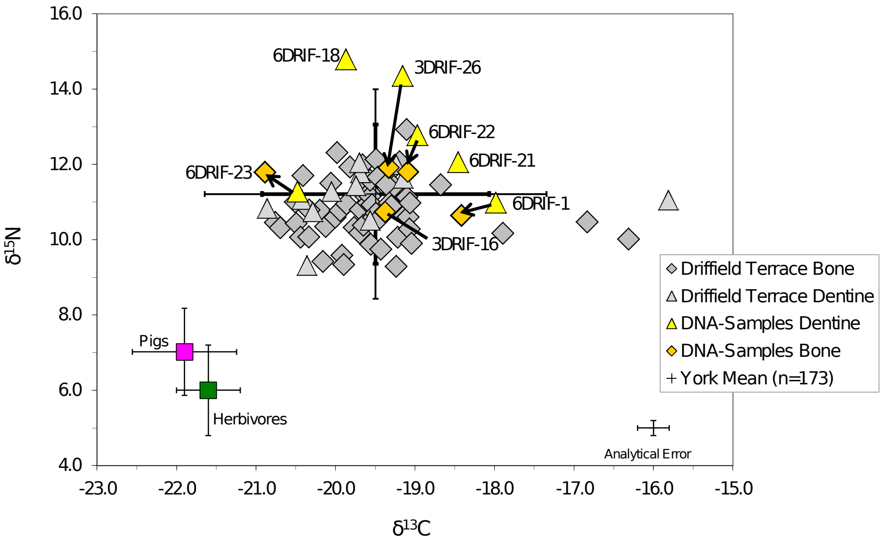

Supplementary Figure 4 - A biplot showing the distribution of strontium isotope and concentration data for archaeological human enamel excavated in Britain for: all periods; the Roman Period; and the seven individuals from Driffield Terrace, York analysed for aDNA. As the data are drawn from multiple sources 1–6 analytical uncertainty is estimated at +/- 0.002 % (2 SD) for 87Sr/86Sr (i.e. within symbol) and +/- 10 mg/kg for strontium concentration.

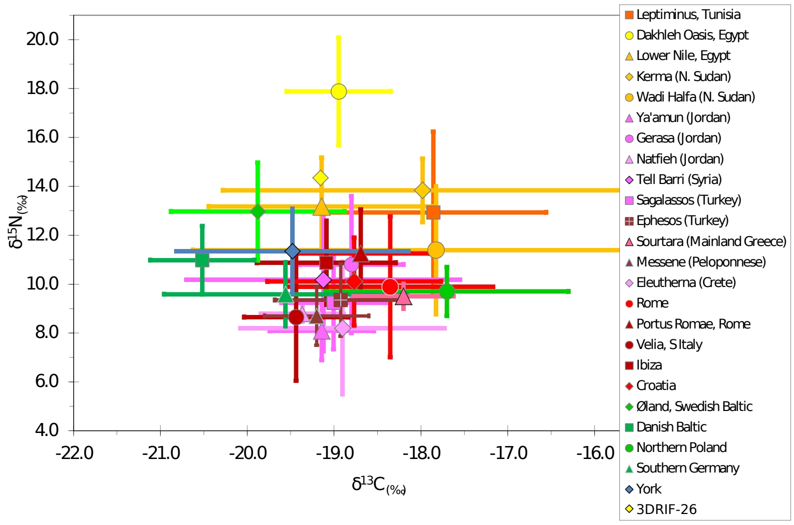

Supplementary Figure 5 - Carbon and nitrogen stable isotope data from Driffield Terrace in comparison with mean values for fauna (±1SD) and humans (stepped error bars: ±2SD and ±3SD) from Roman York. Arrows connect isotope data from dentine (diet in late childhood/adolescence) and bone (adult diet) of the genotyped individuals. Data: 6–8.

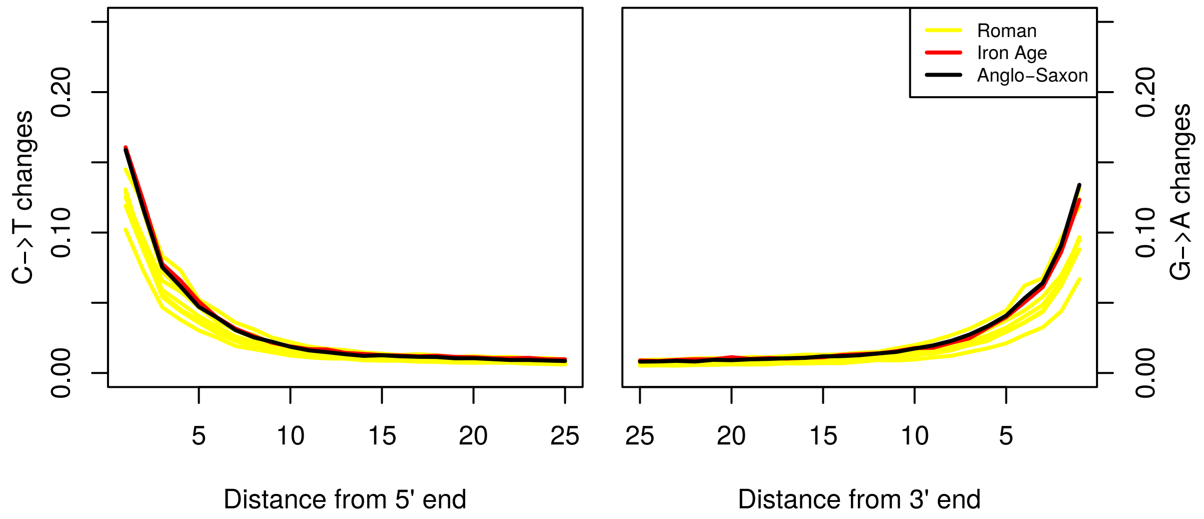

Supplementary Figure 6 - Comparison of aDNA misincorporation patterns at the 5′ end of sequencing reads (C to T) and 3' end (G to A) between the samples from Roman York, Iron Age and Anglo-Saxon.

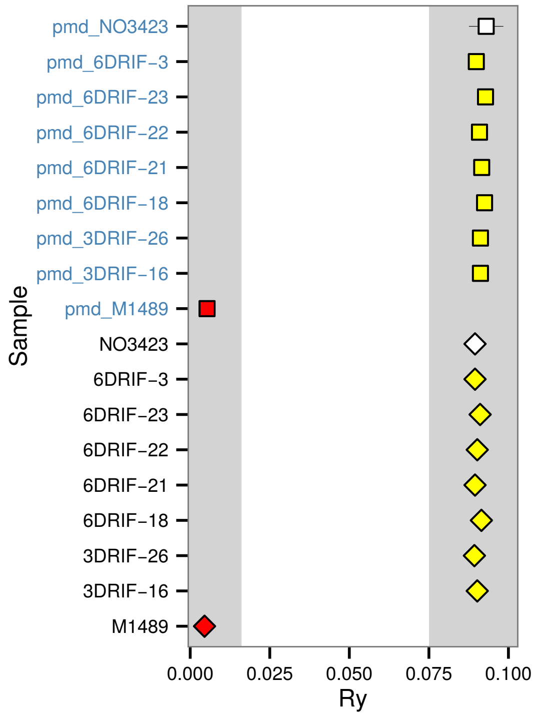

Supplementary Figure 7 - Sex identification based on shotgun sequencing data and comparison of results using reads with evidence of deamination (PMD score 3, squares) and unfiltered reads (lozenges). Ry - Ratio of the number of reads aligned to the Y-chromosome divided by the sum of the number reads aligned to the Y- and X-chromosomes. Gray shaded areas represent threshold for acceptance of assignment, calibrated with modern and ancient genomes. Error bars correspond to 95% confidence intervals.

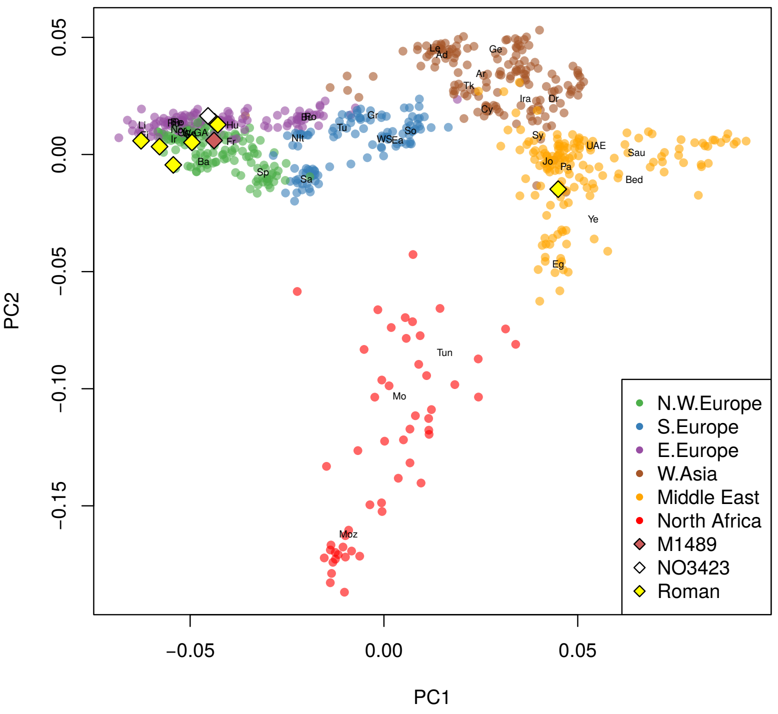

Supplementary Figure 8 - Principal Component Analysis of ancient individuals merged with genotypes from 780 European, West Asian, North African and Middle Eastern individuals using reads with evidence of deamination (PMD score 3).

Population key: Ad - Adygei; Ar - Armenian; Ba - Basque; Bed - Bedouin; Be - Belorussian; Bu - Bulgarian; Ch - Chuvash; Cy - Cypriot; Dr - Druze; Ea - EastSicilian; Eg - Egyptian; En - English; Fi - Finnish; Fr - French; Ge - Georgian; GA - GermanyAustria; Gr - Greek; Hu - Hungarian; Ira - Iranian; Ir - Ireland; Jo - Jordanian; Le - Lezgin; Li - Lithuanian; Mo - Moroccan; Moz - Mozabite; NIt - NorthItalian; Nor - Norwegian; Or - Orcadian; Pa - Palestinian; Po - Polish; Ro - Romanian; Ru - Russian; Sa - Sardinian; Sau - Saudi; Sc - Scottish; So - SouthItalian; Sp - Spanish; Sy - Syrian; Tun - Tunisian; Tk - Turkish; Tu - Tuscan; UAE - UAE; We - Welsh; WS - WestSicilian; Ye - Yemeni.

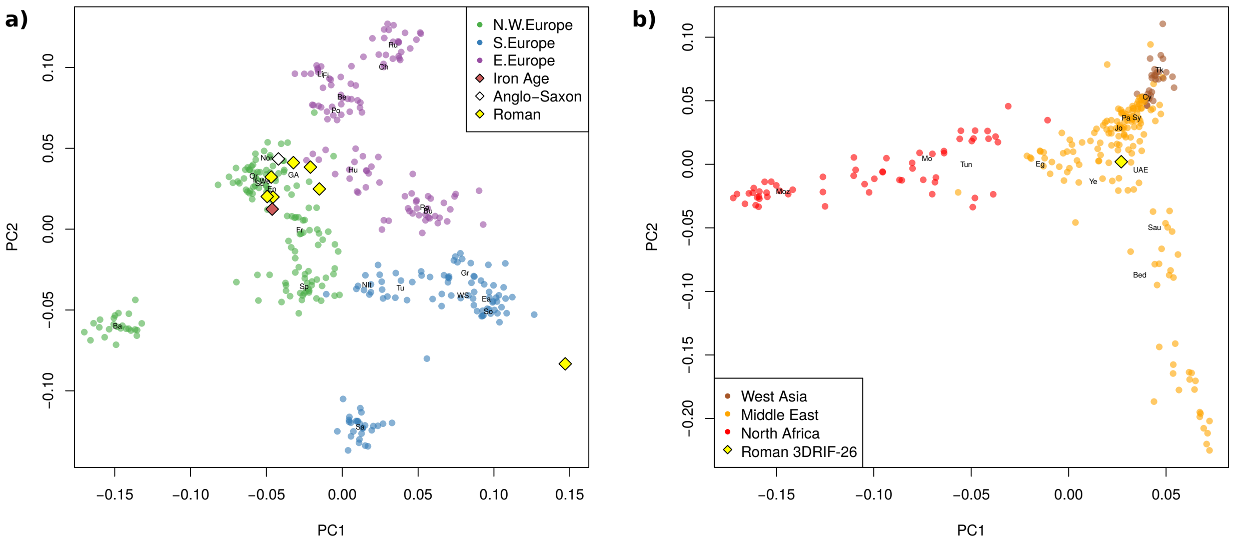

Supplementary Figure 9 - Principal Component Analysis of **a)** ancient individuals merged with genotypes from European populations only. Note that 3DRIF-26 is outside present-day European variation, but in closer proximity to southern European populations. **b)** 3DRIF-26 and Middle Eastern populations.

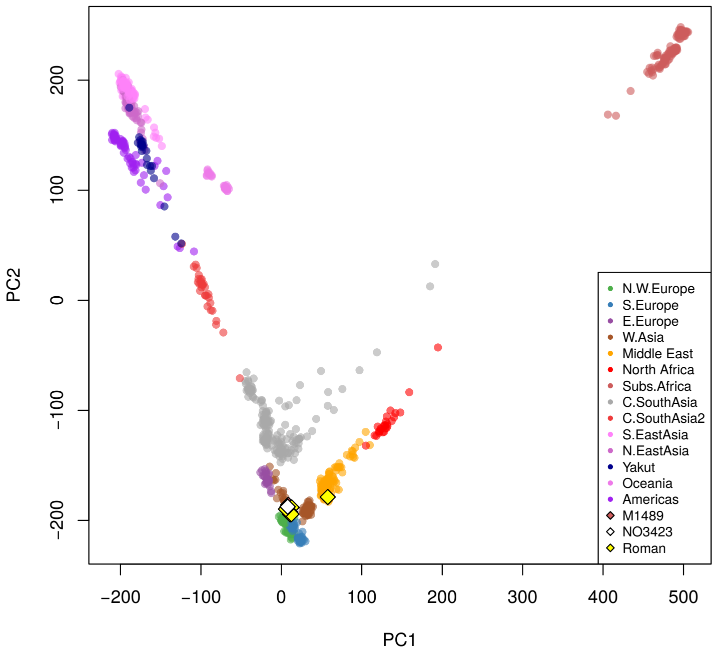

Supplementary Figure 10 - PCA made using LASER. Includes genotype data from ancient samples and present-day individuals from the HGDP.

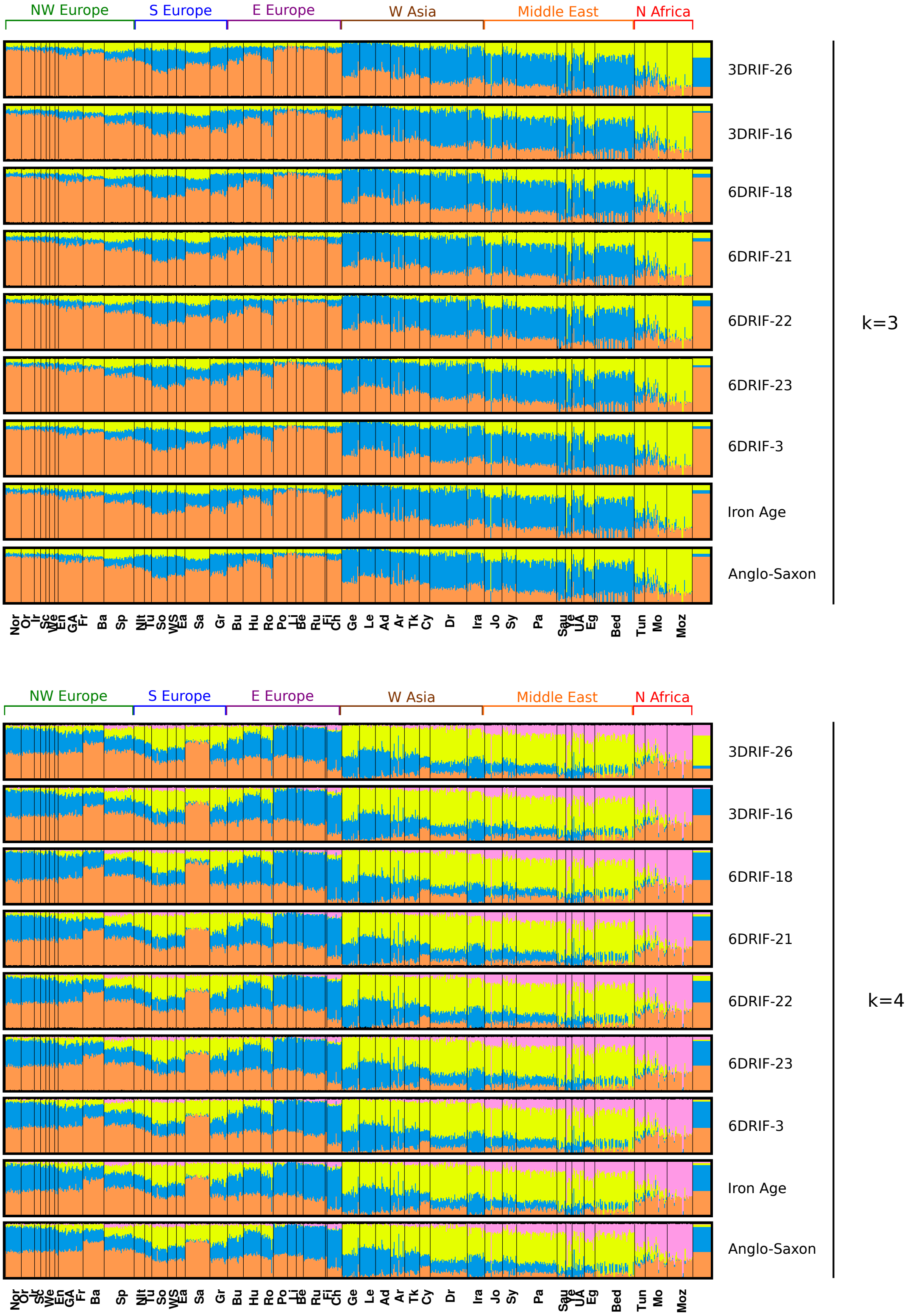

Supplementary Figure 11 - ADMIXTURE analysis of ancient individuals merged separately with genotype data of 780 European, West Asian, North African and Middle Eastern individuals assuming K=3 and K=4.

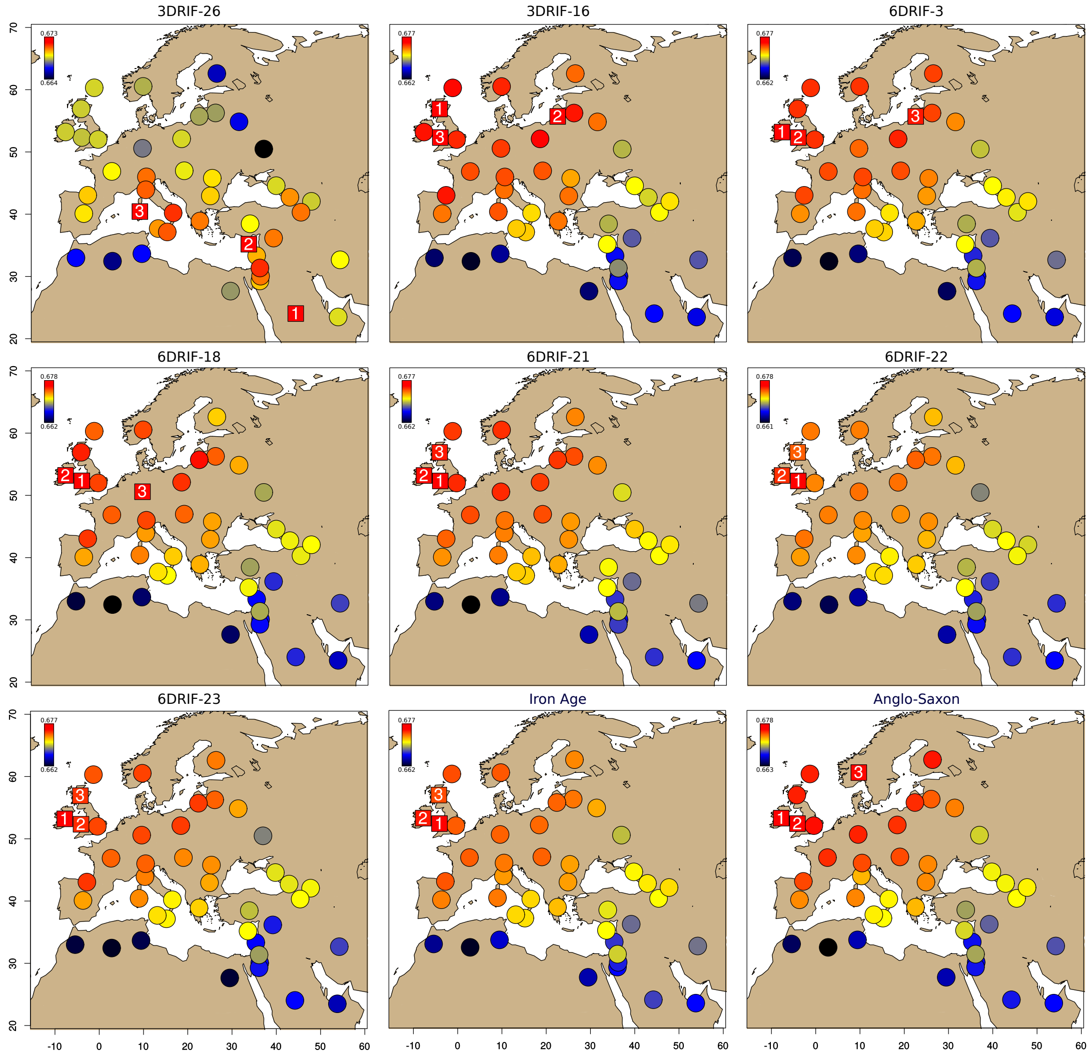

Supplementary Figure 12 - Median proportions of identity-by-state at SNP loci between ancient samples and modern populations. Numbered squares (1-3) represent the 3 best matching populations.

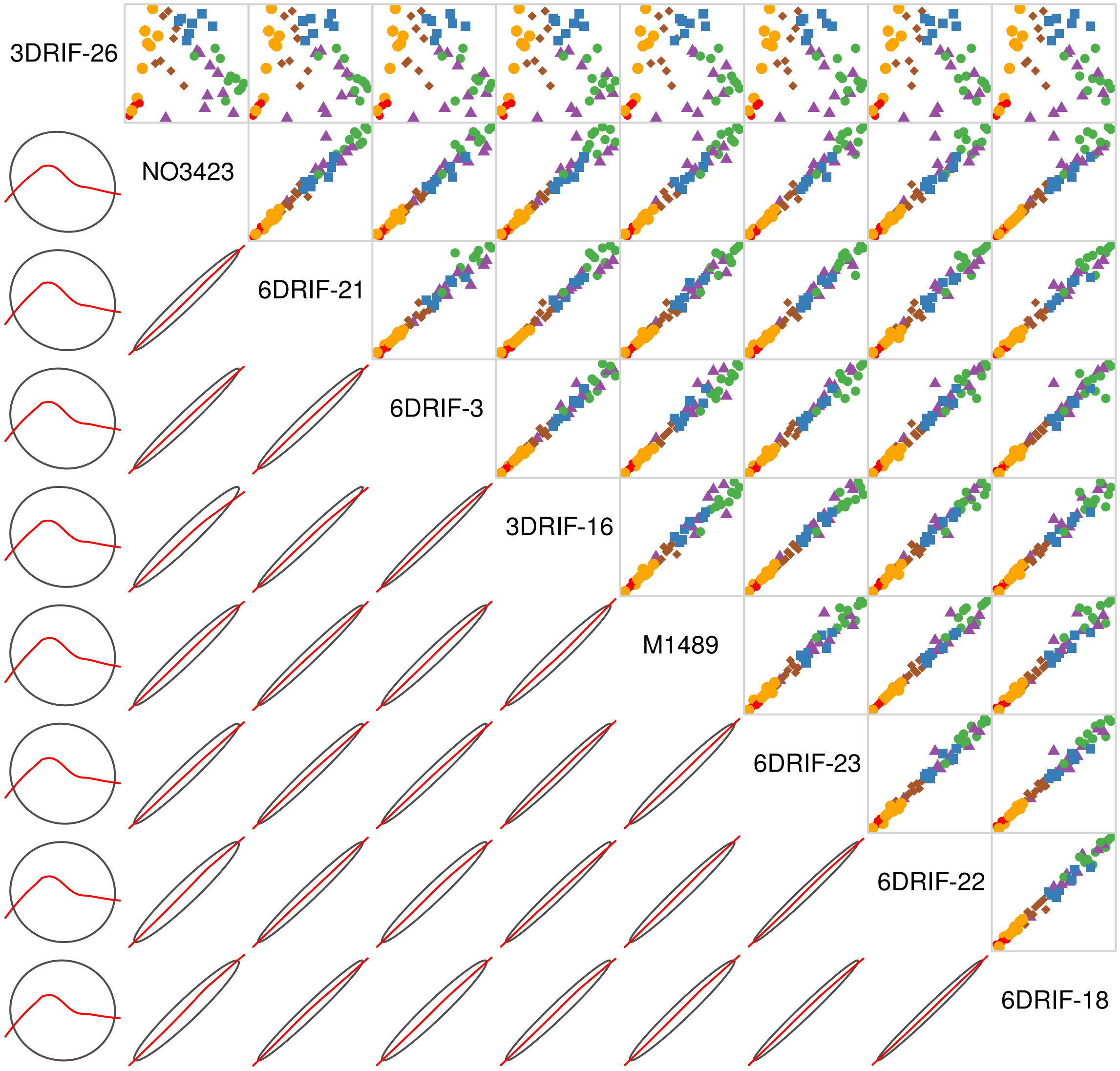

Supplementary Figure 13 - Correlogram illustrating pairwise identity-by-state rank correlations between ancient samples and modern populations. Best fit regression lines are shown on the left-hand side pannel of the plot.

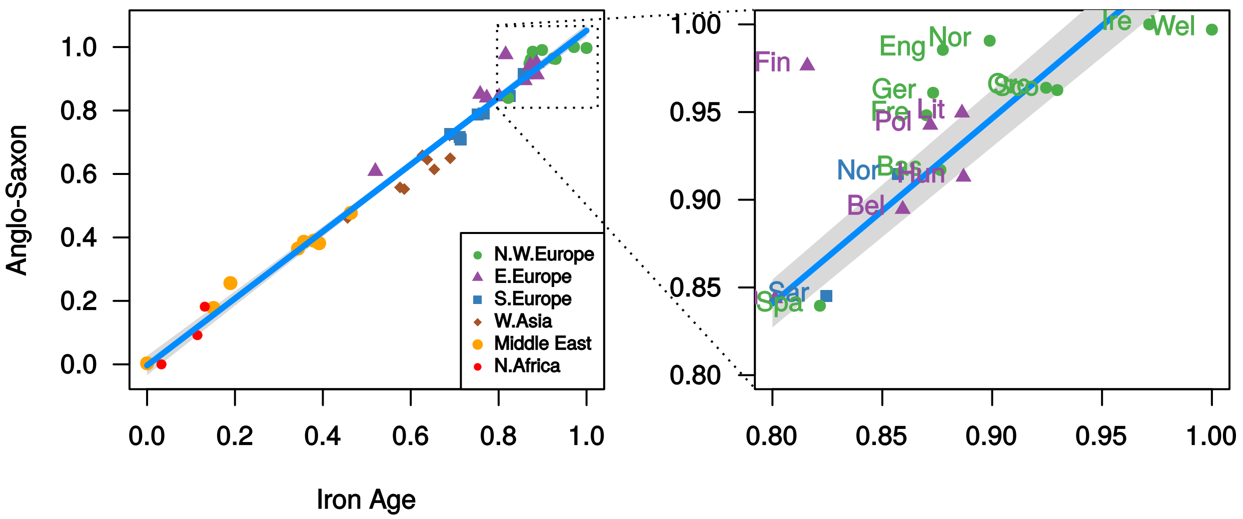

Supplementary Figure 14 - Comparison between Anglo-Saxon and Iron Age IBS with present-day populations. Although IBS profiles appear to be highly correlated, more detailed observation may provide insights into migration movements into Britain across time. While the Anglo-Saxon sample shows greater similarity with Northern European (Norwegian, Finnish), English and German/Austrian samples, the Iron Age appears to be more similar with the Welsh. Despite these differences being very small, the fact that they are concordant across datasets provides further support to the signals observed.

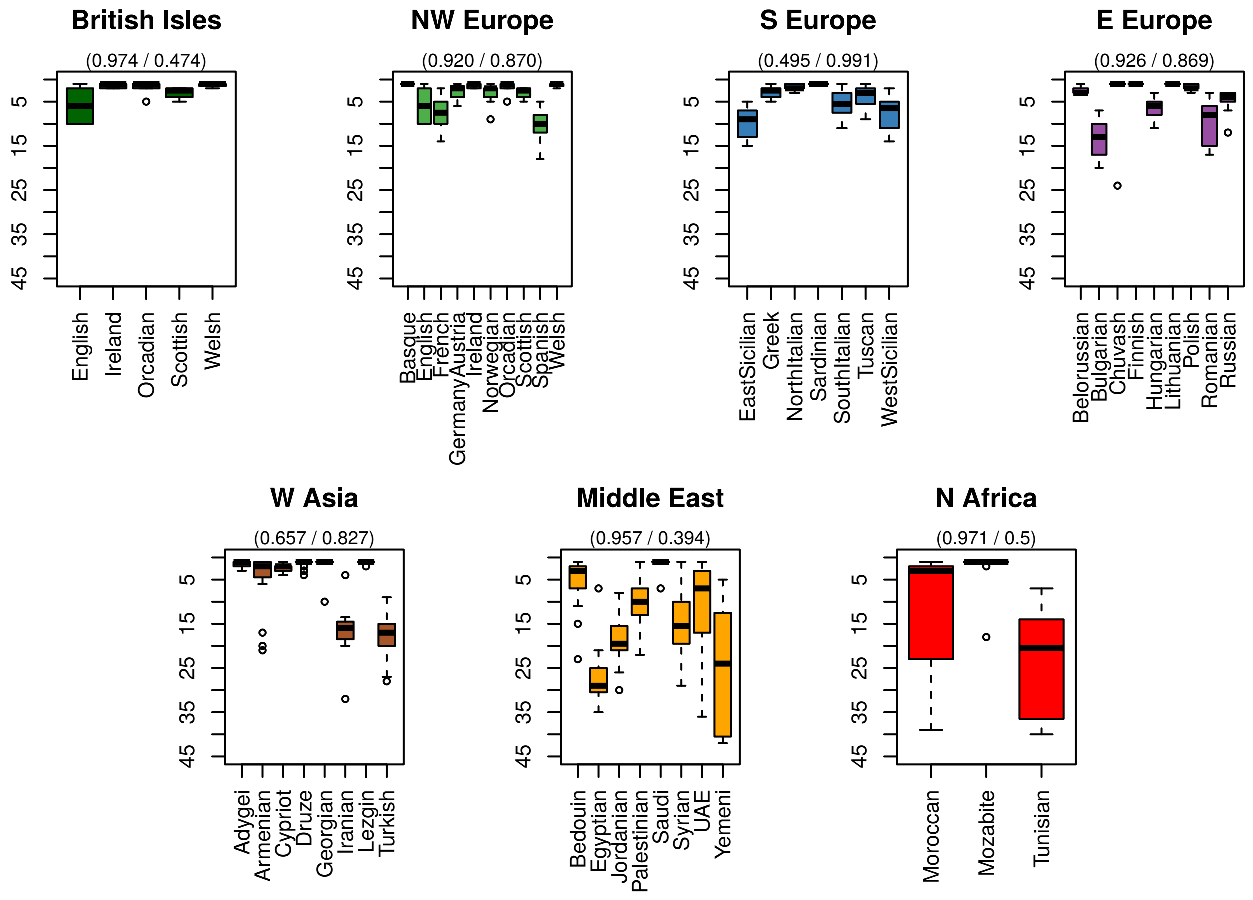

Supplementary Figure 15 - Boxplots showing the rank at which a given individual's own population is positioned in terms of IBS. Figures in brackets represent the specificity of this approach (the proportion of individuals that are correctly assigned to a certain region) and sensitivity (whether modern samples can be assigned to their region of origin).

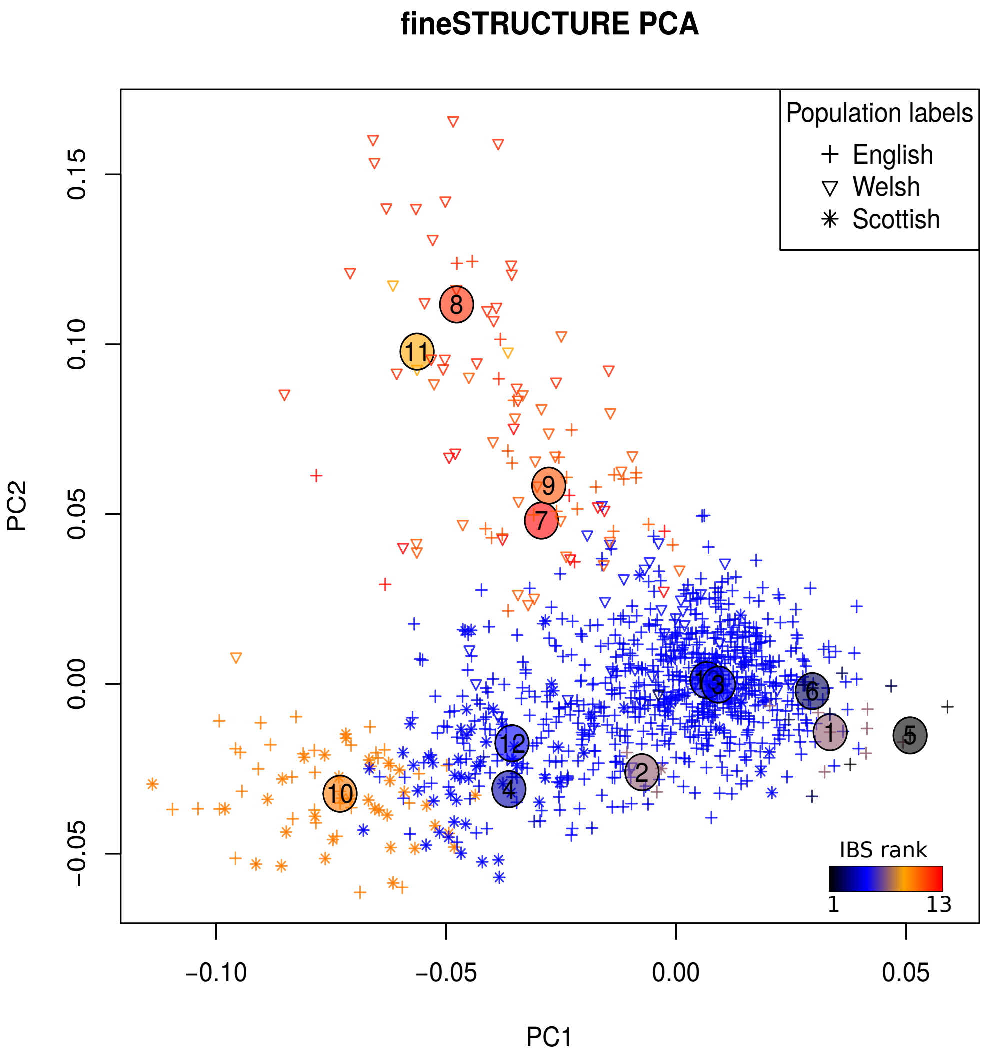

Supplementary Figure 16 - Principal components plot based on the chunkcounts coancestry matrix produced by fineSTRUCTURE on a subset of the WTCCC1 data. Structure is apparent with both Welsh and Scottish samples separating out from a core of English provenance. FineSTRUCTURE cluster centroid values are denoted by circles and individuals from each cluster are coloured according to cluster median IBS values when compared to the six Roman samples. Welsh samples show closest affinity to the ancients.

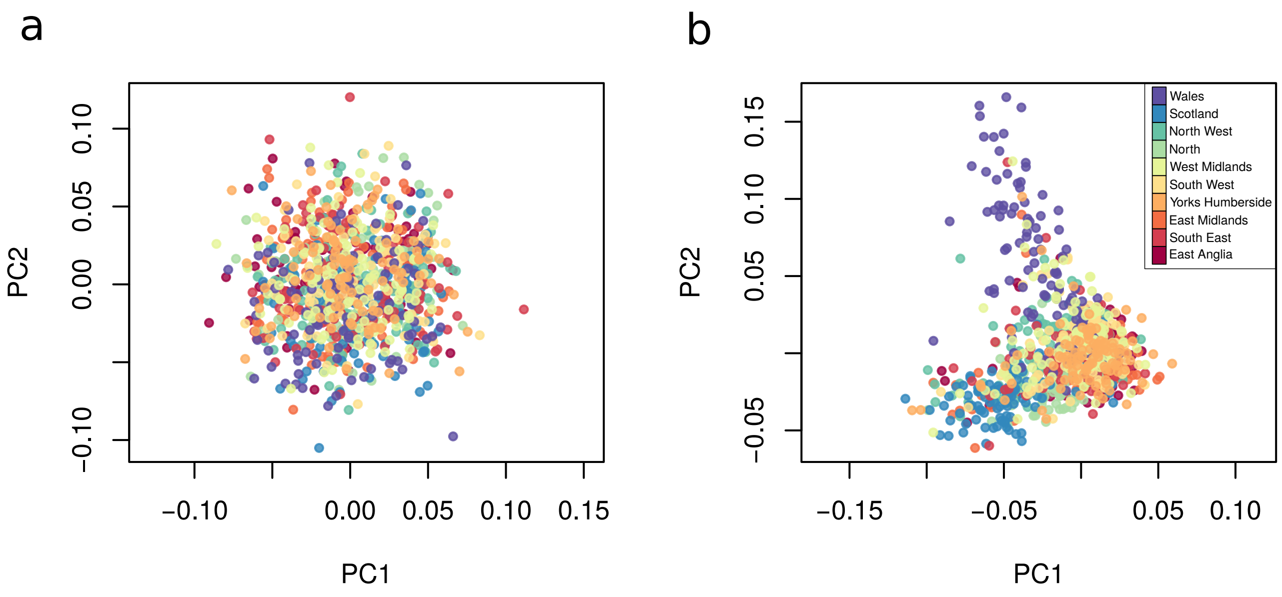

Supplementary Figure 17 - Principal components plot based on a) a subset of WTCCC1 genotype data and b) the chunkcounts coancestry matrix produced by fineSTRUCTURE. Individual points are coloured according to geographical region. The advantage of using haplotype based methods to infer population structure is evident.

Supplementary Tables

Supplementary Table 1 - Age and sex of skeletons from Driffield Terrace.

Age Group	Male n	Female n	Unsexed n	Total n
f	-	-	1	1
n	-	-	1	1
i	-	-	0	0
j	-	-	2	2
ad	-	-	3	3
ya	16	0	1	17
yma	27	1	0	28
oma	19	0	0	19
ma	0	0	0	0
a	4	0	7	11
Total	66	1	15	82

f = foetus (<38 weeks *in utero*); n = neonate (0-1 month); i = infant (1-12 months); j = juvenile (1-12 years); ad = adolescent (13-17 years); ya = young adult (18-25 years); yma = young middle adult (26-35 years); oma = old middle adult (36-45 years); ma = mature adult (46+ years); a = adult (18+ years)

Supplementary Table 2 - Osteological and palaeopathological data for sampled skeletons.

Sk No	Sex	Age Group	Osteology
M1489 (Iron Age)	F	YMA	Joint disease (spine & L TMJ); Schmorl's nodes; Cribra orbitalia; Bipartite/fractured foot sesamoid bone; Calculus; DEH; Notch between upper central incisors
3DRIF-16	M	OMA	Sacralisation of L6; Fracture of left distal radius (wrist); Osteochondritis dissecans; L tibia; Fracture T4 spinous process; Joint disease; Maxillary sinusitis; Fractured lower L canine; Dental calculus; Caries; DEH; PD
3DRIF-26	M?	OMA	Spondylolysis & spondylolisthesis; cleft arches L5, S1-5; Fractured transverse processes, L2 & L3; ? Infection of left ulna; Trauma to L shoulder (probable reduced dislocation); Gracile left arm; Un-united fracture of L scaphoid; Joint disease; Fractured R rib 10; Cribra orbitalia; Lamellar bone on tibiae; AMTL; calculus; DEH; PD; notch in upper LI1
6DRIF-3	M	YA	Supernumerary tooth; Os tibiale externum/ avulsion fracture (R navicular); ?Avulsion fracture of Right MC3; Cribra orbitalia; Calculus; DEH; Notch in incisal surface upper RI1 and lower RI1; Peri-mortem butterfly fracture of R ulna
6DRIF-18	M	YA	Cribra orbitalia; Clay shoveller's fracture of T1; Calculus; DEH
6DRIF-21	M	OMA	Fracture of distal R tibia; Fracture of left fibula & soft tissue trauma to left tibia; Avulsion fracture of R hamate; Lytic area in bodies L1 & L2; Cribra orbitalia; Maxillary sinusitis; Os acromiale; Avulsion fracture of R ulna; Lamellar bone on both tibiae, right ribs 11 & 12; Calculus; Caries; DEH; Abscess; PD
6DRIF-22	M	YMA	?Penetrating injury to left occipital; ?Stab to neck; Lamellar bone on L & R ribs 11 & 12; Caries; Abscess; Cyst in left femur; Joint disease; Cribra orbitalia; Maxillary sinusitis; Additional thoracic vertebra; Ivory osteoma; AMTL; Calculus; PD
6DRIF-23	M	YA	Cut to R mandibular ramus; Four cuts to C2-5 & mandible; full decapitation at C5 Calculus; PD
NO3423 (Anglo-Saxon)			Norton on Tees Skeleton

Age Group: YA = young adult (18-25 years); YMA = young middle adult (26-35 years); OMA = old middle adult (36-45 years); MA = mature adult (46+ years)

DEH = dental enamel hypoplasia; PD = periodontal disease; AMTL = ante-mortem tooth loss

Supplementary Table 3 - Carbon, oxygen and strontium isotope data and concentrations for individual 3DRIF-26 and conversions from oxygen isotope ratios of structural carbonate (δ18Oc) to phosphate (δ18Op) and drinking water values (δ18Odw).

**Sample No.**	**Tooth**	**Tissue**	**13C(VPDB)**	**18OC (VSMOW)**	**18Op (VSMOW)1**	**18Odw2**	**18Op (VSMOW)3**	**18Odw4**	**Sr**	**87Sr/86Sr5**
			**measured ‰**	**measured ‰**	**calculated ‰**	**calculated ‰**	**calculated ‰**	**calculated ‰**	**measured ‰**	**measured ‰**
3DRIF-26	Left mandibular PM2	enamel	-12.6±0.1	31.6±0.1	22.9	1.6	21.7	-0.3	177	0.70777

1 calculated using[51]; 2 calculated using[52], Equation 6; 3 calculated using[10]; 4 calculated using[10,52], Equation 6.

Supplementary Table 4 - Carbon and nitrogen stable isotope data for sequenced individuals from Driffield Terrace.

**Sample No.**	**Element**	**Tissue**	**13C(VPDB) ‰**	**15N(AIR)‰**	**%C**	**%N**	**C/N**	**%Coll**
3DRIF-16 [1]	Rib	bone	-19.4	10.7	35.7	12.8	3.3	3.5
3DRIF-26	Left mandibular PM2	dentine, root mid-section	-19.2	14.3	43.4	15.7	3.2	16.1
3DRIF-26 [1]	rib	bone	-19.3	11.9	35.5	12.7	3.3	7.3
6DRIF-1 [1,2]	Left maxillary PM2	dentine, root mid-section	-18	11	43.9	15.9	3.2	16
6DRIF-3 [2]	Right ulna (distal)	bone	-18.4	10.6	33.4	12.1	3.2	5.6
6DRIF-18T-1 [1]	Left maxillary PM2	dentine, root at dentino-enamel junction	-19.9	14.8	41.3	15.1	3.2	8.5
6DRIF-18T-2	Left maxillary PM2	dentine, root mid-section	-20.1	14.3	45	15.9	3.3	9.4
6DRIF-18T-3	Left maxillary PM2	dentine, root tip	-19.9	14.7	44.9	16	3.3	10.4
6DRIF-18 1	Rib	bone	--	--	--	--	--	Nil
6DRIF-21 [1]	Right maxillary PM2	dentine	-18.5	12.1	42.1	15.4	3.2	16
6DRIF-21 [3]	Right radius (shaft)	bone	-19.8	11.9	38	12.4	3.6	0.4
6DRIF-22 [1]	Left mandibular PM2	dentine	-19	12.8	41.5	15.3	3.2	14.3
6DRIF-22	Right clavicle (lateral shaft)	bone	-19.1	11.8	35.2	12.2	3.4	1.4

1 data published in[6];

2 cranium 6DRIF-1 and post-cranial skeleton 6DRIF-3 belong to the same individual;

3 excluded on grounds of poor yield and marginal C/N ratio.

Supplementary Table 5 - Mitochondrial DNA contamination estimates.

Sample	Contamination %	Contamination % *
3DRIF-16	1.7	1.24
3DRIF-26	1.54	0.88
6DRIF-18	1.27	0.85
6DRIF-21	1.62	1.09
6DRIF-22	2.09	1.43
6DRIF-23	2.3	1.63
6DRIF-3	1.26	1.03
Iron Age (M1489)	2.69	2.42
Anglo-Saxon (NO3423)	1.88	1.3

*after mapDamage rescaling.

Supplementary Table 6 - X chromosome Contamination estimates for method 1 and method 2 performed as in [36]).

	Method 1			Method 2		
Sample	Contamination %	SD	P-value	Contamination %	SD	P-value
3DRIF-16	0.78	0.31	0.02519	1.33	0.53	0.01004
3DRIF-26	0.79	0.17	3.14E-06	0.77	0.25	0.00142
6DRIF-18	0.69	0.17	1.57E-06	0.68	0.24	0.003813
6DRIF-21	0.66	0.16	3.22E-05	0.43	0.22	0.01192
6DRIF-22	0.73	0.17	8.39E-06	0.78	0.25	0.00186
6DRIF-23	0.85	0.27	0.005495	0.62	0.36	0.1875
6DRIF-3	0.67	0.12	1.82E-10	0.73	0.19	9.70E-08
Anglo-Saxon (NO3423)	0.93	0.19	3.89E-05	1.18	0.29	0.004054

Supplementary Table 7 - Major and minor alleles at SNP sites and adjacent bases on the X-chromosome.

					Method 1					
Sample	Base	-4	-3	-2	-1	SNP site	1	2	3	4
3DRIF-16	Minor base	15	23	21	31	36	23	34	21	21
	Major base	6132	6.12E+03	6128	6121	6121	6119	6112	6129	6115
3DRIF-26	Minor base	66	7.50E+01	77	69	119	78	59	87	72
	Major base	18501	1.85E+04	18500	18507	18413	18483	18522	18468	18499
6DRIF-18	Minor base	27	2.40E+01	27	30	61	41	31	24	29
	Major base	15092	15087	15092	15091	15068	15076	15106	15124	15094
6DRIF-21	Minor base	35	5.10E+01	30	51	8.00E+01	55	53	48	48
	Major base	17463	1.74E+04	17453	17463	17421	17444	17436	17446	17465
6DRIF-22	Minor base	54	40	39	53	80	43	40	40	48
	Major base	16213	16199	16222	16222	16211	16262	16266	16263	16232
6DRIF-23	Minor base	17	6	22	22	32	20	17	19	22
	Major base	6359	6381	6370	6369	6356	6369	6372	6376	6368
6DRIF-3	Minor base	107	91	113	123	180	92	92	104	105
	Major base	32919	32956	32947	32900	32834	32938	32949	32947	32920
NO3423	Minor base	54	55	48	70	98	71	70	51	66
	Major base	15034	15067	15085	15060	15038	15052	15039	15057	15004
					Method 2					
Sample	Base	-4	-3	-2	-1	SNP site	1	2	3	4
3DRIF-16	Minor base	5	12	11	12	20	10	15	9	7
	Major base	2830	2823	2824	2823	2815	2825	2820	2826	2828
3DRIF-26	Minor base	31	33	27	27	51	36	24	32	32
	Major base	7969	7967	7973	7973	7949	7964	7976	7968	7968
6DRIF-18	Minor base	13	13	13	7	25	14	12	12	16
	Major base	6663	6663	6663	6669	6651	6662	6664	6664	6660
6DRIF-21	Minor base	14	27	12	27	31	18	14	22	14
	Major base	7630	7617	7632	7617	7613	7626	7630	7622	7630
6DRIF-22	Minor base	19	15	19	22	34	15	17	21	18
	Major base	7090	7094	7090	7087	7075	7094	7092	7088	7091
6DRIF-23	Minor base	5	3	6	13	12	10	7	8	9
	Major base	2960	2962	2959	2952	2953	2955	2958	2957	2956
6DRIF-3	Minor base	49	28	42	46	79	28	34	33	48
	Major base	13495	13516	13502	13498	13465	13516	13510	13511	13496
Anglo-Saxon (NO3423)	Minor base	23	21	18	28	40	27	27	19	25
	Major base	6603	6605	6608	6598	6586	6599	6599	6607	6601

Supplementary Table 8 - Qubit DNA quantification of DNA samples and controls

Sample	ug/mL
3DRIF-16	6.27
3DRIF-26	3.66
6DRIF-18	5.59
6DRIF-21	8.56
6DRIF-22	9.44
6DRIF-23	11.3
6DRIF-3	11.1
M1489	19.1
NO3423	29.3
Extraction control	-
Library control	-
PCR control	-

Supplementary Table 9- Mitochondrial DNA haplogroups and mutations identified.

Sample	Haplogroup	Average Coverage	Polymorphisms
3DRIF-16	H6a1a	75.67	73A 146T 152T 195T 239C 247G 769G 825T 1018G 2706A 2758G 2885T 3548C 3594C 3915A 4104A 4312C4727G 7028C 7146A 7256C 7521G 8468C 8655C 8701A 9380A 9540T 10398A 10664C 10688G 10810T 10873T 10915T 11253C 11719G 11914G 12705C 13105A 13276A 13506C13650C 14766C 16129G 16187C 16189T 16223C 16230A 16278C 16311T 16362C 16482G 16519T
3DRIF-26	H5	86.95	73A 146T 195T 247G 456T 769G 825T 1018G 2706A 2758G 2885T 3594C 4104A 4312C 5349T 6041T 7028C7146A 7256C 7521G 8468C 8655C 8701A 9540T 10398A 10664C 10688G 10810T 10873T 10915T 11719G 11914G 12705C 13105A 13276A 13506C 13650C 14766C 16129G 16187C16189T 16223C 16230A 16278C 16304C 16311T 16519T
6DRIF-18	H1bs	74.2	73A 146T 152T 195T 247G 769G 825T 1018G 2706A 2758G 2885T 3010A 3594C 4104A 4312C 7028C 7146A7256C 7521G 8468C 8655C 8701A 9540T 10398A 10664C 10688G 10810T 10873T 10915T 11719G 11914G 12705C 13105A 13276A 13506C 13650C 14766C 16129G 16187C 16189T16220C 16223C 16230A 16278C 16311T
6DRIF-21	J1c3e2	79.41	146T 152T 185A 195T 228A 247G 295T 462T 489C 769G 825T 1018G 2758G 2885T 3010A 3594C 4104A4216C 4312C 7146A 7256C 7521G 8468C 8655C 8701A 8865A 9540T 9957C 10664C 10688G 10810T 10873T 10915T 11251G 11914G 12612G 12705C 13105A 13276A 13506C13650C 13708A 13934T 14798C 15452A 16069T 16126C 16129G 16187C 16189T 16223C 16230A 16278C 16311T 16390A 16519T
6DRIF-22	H	78.61	73A 146T 152T 247G 769G 825T 1018G 2706A 2758G 2885T 3594C 4104A 4312C 7028C 7146A 7256C 7521G8468C 8655C 8701A 9540T 10398A 10664C 10688G 10810T 10873T 10915T 11719G 11914G 12705C 13105A 13276A 13506C 13650C 14766C 16129G 16187C 16189T 16223C 16230A16278C 16569del
6DRIF-23	H6a1b2	58.33	73A 146T 152T 195T 239C 247G 769G 825T 1018G 2706A 2758G 2885T 3594C 3915A 4104A 4312C 4727G7028C 7146A 7256C 7521G 8468C 8655C 8701A 9254G 9380A 9540T 10398A 10589A 10664C 10688G 10810T 10873T 10915T 11719G 11914G 12705C 13105A 13276A 13506C13650C 14766C 16129G 16187C 16189T 16212G 16223C 16230A 16278C 16311T 16362C 16482G 16519T
6DRIF-3	J1b1a1	98.07	152T 195T 242T 247G 295T 462T 489C 769G 825T 1018G 2158C 2758G 2885T 3010A 3594C 4104A 4216C4312C 5460A 7146A 7256C 7521G 8269A 8468C 8557A 8655C 8701A 9540T 10664C 10688G 10810T 10873T 10915T 11251G 11914G 12007A 12612G 12705C 13105A 13276A13506C 13650C 13708A 13879C 15452A 16069T 16126C 16129G 16145A 16172C 16187C 16189T 16222T 16223C 16230A 16261T 16278C 16286T 16311T 16519T
Iron Age (M1489)	U2e1e	39.01	146T 195T 217C 247G 340T 508G 769G 825T 1018G 1811G 2526T 2758G 2885T 3594C 3720G 4104A 4312C5390G 5426C 6045T 6152C 7146A 7256C 7521G 8468C 8655C 8701A 9540T 10398A 10664C 10688G 10810T 10873T 10876G 10915T 11467G 11914G 12308G 12372A 12618A12705C 13020C 13105A 13276A 13506C 13650C 13734C 15907G 16051G 16129C 16145A 16187C 16189T 16223C 16230A 16278C 16311T 16362C 16390A 16568_16569del
Anglo-Saxon (NO3423)	H1a	88.44	146T 152T 195T 247G 769G 825T 1018G 2706A 2758G 2885T 3010A 3594C 4104A 4312C 7028C 7146A 7256C7521G 8468C 8655C 8701A 9540T 10398A 10664C 10688G 10810T 10873T 10915T 11719G 11914G 12705C 13105A 13276A 13506C 13650C 14766C 16129G 16162G 16187C 16189T16223C 16230A 16278C 16311T

Supplementary Table 10- Y-chromosome DNA haplogroups and derived alleles identified.

Sample	Chromosome	Position	Marker	Haplogroup	Mutation	Ancestral	Reads	Derived
3DRIF-T16	chrY	8050994	P229	R	G->C	G	1	C
	chrY	18914441	L278	R1b1	C->T	C	1	T
	chrY	22739367	M269	R1b1a2	T->C	T	1	C
	chrY	8796078	M405/S21	R1b1a2a1a1	C->T	C	1	T
3DRIF-26	chrY	8590752	P127	IJ	C->T	C	1	T
	chrY	7292720	S34	J	A->G	T	1	C
	chrY	8418927	S35	J	G->C	G	3	C
	chrY	7771358	L228	J2	C->T	C	1	T
6DRIF-18	chrY	8050994	P229	R	G->C	G	1	C
	chrY	7570822	P294	R1	G->C	G	2	C
	chrY	7647357	P242	R1	G->A	G	1	A
	chrY	18656508	P297	R1b1a	G->C	G	3	C
	chrY	22739367	M269	R1b1a2	T->C	T	1	C
	chrY	2842212	L49.1	R1b1a2a	T->A	T	1	A
	chrY	8502236	L51	R1b1a2a1	G->A	G	2	A
	chrY	14641193	L52	R1b1a2a1a	C->T	C	1	T
	chrY	17844018	L11/S127	R1b1a2a1a	T->C	T	1	C
6DRIF-21	chrY	6868118	S9	R	T->C	G	2	A
	chrY	8050994	P229	R	G->C	G	1	C
	chrY	7570822	P294	R1	G->C	G	1	C
	chrY	18914441	L278	R1b1	C->T	C	2	T
	chrY	22739367	M269	R1b1a2	T->C	T	1	C
	chrY	2842212	L49.1	R1b1a2a	T->A	T	1	A
	chrY	8502236	L51	R1b1a2a1	G->A	G	1	A
	chrY	14641193	L52	R1b1a2a1a	C->T	C	1	T
	chrY	17844018	L11/S127	R1b1a2a1a	T->C	T	1	C
	chrY	22157311	P312	R1b1a2a1a2	C->A	C	1	A
	chrY	2883986	DF63	R1b1a2a1a2c2	T->C	T	2	C
6DRIF-22	chrY	7570822	P294	R1	G->C	G	1	C
	chrY	18914441	L278	R1b1	C->T	C	1	T
	chrY	18656508	P297	R1b1a	G->C	G	1	C
	chrY	22739367	M269	R1b1a2	T->C	T	2	C
	chrY	8502236	L51	R1b1a2a1	G->A	G	1	A
	chrY	17844018	L11/S127	R1b1a2a1a	T->C	T	1	C
	chrY	22157311	P312	R1b1a2a1a2	C->A	C	1	A
	chrY	15333149	S28; U152	R1b1a2a1a2b	C->T	C	1	T
6DRIF-23	chrY	6868118	S9	R	T->C	G	1	A
	chrY	22739367	M269	R1b1a2	T->C	T	1	C
	chrY	14641193	L52	R1b1a2a1a	C->T	C	2	T
6DRIF-3	chrY	6868118	S9	R	T->C	G	3	A
	chrY	7570822	P294	R1	G->C	G	1	C
	chrY	18914441	L278	R1b1	C->T	C	1	T
	chrY	22739367	M269	R1b1a2	T->C	T	2	C
	chrY	2668456	PF6399	R1b1a2	C->T	C	1	T
	chrY	14641193	L52	R1b1a2a1a	C->T	C	1	T
	chrY	8796078	M405/S21	R1b1a2a1a1	C->T	C	1	T
Anglo-Saxon (NO3423)	chrY	7173143	L16	IJK	G->A	G	1	A
	chrY	3545070	P212	I	T->A	T	1	A
	chrY	6677619	S107	I1	A->C	T	1	G

Supplementary Table 11 - Median identity-by-state between ancient samples and modern populations.

Population	Iron Age	3DRIF-16	3DRIF-26	6DRIF-18	6DRIF-21	6DRIF-22	6DRIF-23	6DRIF-3	Anglo-Saxon
Adygei	0.672	0.672	0.670	0.672	0.673	0.671	0.671	0.672	0.673
Armenian	0.672	0.672	0.672	0.672	0.672	0.672	0.672	0.671	0.673
Basque	0.676	0.677	0.671	0.676	0.675	0.676	0.676	0.676	0.677
Bedouin	0.667	0.667	0.672	0.667	0.667	0.668	0.666	0.666	0.668
Belorussian	0.675	0.677	0.669	0.676	0.675	0.676	0.675	0.676	0.676
Bulgarian	0.674	0.675	0.671	0.674	0.674	0.674	0.673	0.674	0.676
Chuvash	0.670	0.671	0.664	0.671	0.671	0.670	0.669	0.671	0.672
Cypriot	0.672	0.672	0.673	0.672	0.671	0.673	0.672	0.672	0.672
Druze	0.671	0.670	0.673	0.671	0.670	0.670	0.670	0.670	0.671
EastSicilian	0.673	0.673	0.672	0.673	0.673	0.673	0.672	0.673	0.674
Egyptian	0.664	0.664	0.669	0.664	0.664	0.664	0.663	0.663	0.666
English	0.676	0.676	0.670	0.676	0.676	0.675	0.675	0.676	0.678
Finnish	0.675	0.675	0.666	0.674	0.674	0.674	0.674	0.676	0.678
French	0.675	0.676	0.670	0.676	0.675	0.675	0.675	0.675	0.677
Georgian	0.673	0.671	0.672	0.672	0.672	0.672	0.671	0.672	0.673
GermanyAustria	0.675	0.676	0.669	0.677	0.676	0.676	0.675	0.675	0.677
Greek	0.674	0.675	0.672	0.674	0.673	0.674	0.673	0.673	0.675
Hungarian	0.676	0.675	0.670	0.675	0.675	0.675	0.674	0.675	0.677
Iranian	0.669	0.669	0.670	0.669	0.669	0.668	0.668	0.669	0.670
Ireland	0.677	0.677	0.670	0.677	0.676	0.677	0.677	0.677	0.678
Jordanian	0.667	0.668	0.671	0.668	0.668	0.668	0.667	0.667	0.669
Lezgin	0.673	0.673	0.670	0.672	0.672	0.671	0.671	0.672	0.674
Lithuanian	0.676	0.677	0.669	0.677	0.676	0.676	0.675	0.677	0.677
Moroccan	0.664	0.664	0.667	0.663	0.664	0.663	0.663	0.663	0.664
Mozabite	0.662	0.663	0.666	0.662	0.662	0.663	0.662	0.662	0.663
NorthItalian	0.675	0.676	0.672	0.676	0.674	0.675	0.675	0.676	0.677
Norwegian	0.676	0.676	0.669	0.676	0.676	0.675	0.675	0.676	0.678
Orcadian	0.676	0.677	0.670	0.676	0.676	0.675	0.675	0.676	0.678
Palestinian	0.668	0.668	0.671	0.668	0.668	0.668	0.667	0.668	0.669
Polish	0.675	0.677	0.670	0.676	0.676	0.676	0.675	0.676	0.677
Romanian	0.674	0.674	0.671	0.674	0.674	0.675	0.674	0.674	0.676
Russian	0.674	0.675	0.667	0.674	0.674	0.674	0.673	0.674	0.676
Sardinian	0.675	0.675	0.673	0.675	0.674	0.675	0.674	0.675	0.676
Saudi	0.668	0.668	0.673	0.668	0.668	0.669	0.667	0.667	0.669
Scottish	0.676	0.677	0.670	0.677	0.676	0.676	0.676	0.676	0.678
SouthItalian	0.673	0.673	0.673	0.674	0.673	0.673	0.672	0.672	0.674
Spanish	0.675	0.675	0.671	0.674	0.674	0.675	0.673	0.674	0.676
Syrian	0.669	0.669	0.672	0.668	0.669	0.669	0.667	0.669	0.670
Tunisian	0.664	0.665	0.667	0.664	0.664	0.664	0.663	0.664	0.666
Turkish	0.671	0.671	0.670	0.671	0.671	0.671	0.670	0.670	0.671
Tuscan	0.674	0.675	0.672	0.675	0.674	0.675	0.674	0.675	0.675
UAE	0.665	0.666	0.670	0.666	0.665	0.666	0.665	0.666	0.667
Welsh	0.677	0.677	0.669	0.678	0.677	0.678	0.676	0.677	0.678
WestSicilian	0.673	0.673	0.672	0.673	0.673	0.673	0.672	0.673	0.674
Yemeni	0.662	0.662	0.666	0.663	0.662	0.661	0.662	0.662	0.663

Supplementary Table 12 - Pairwise identity-by-state rank correlations between Roman York samples.

Sample	6DRIF-23	6DRIF-22	6DRIF-21	6DRIF-18	6DRIF-3	3DRIF-26	3DRIF-16
6DRIF-23	1.000	0.990	0.980	0.990	0.980	-0.050	0.990
6DRIF-22	0.990	1.000	0.980	0.990	0.970	-0.020	0.980
6DRIF-21	0.980	0.980	1.000	0.980	0.980	-0.090	0.980
6DRIF-18	0.990	0.990	0.980	1.000	0.970	-0.020	0.980
6DRIF-3	0.980	0.970	0.980	0.970	1.000	-0.080	0.990
3DRIF-26	-0.050	-0.020	-0.090	-0.020	-0.080	1.000	-0.070
3DRIF-16	0.990	0.980	0.980	0.980	0.990	-0.070	1.000

Supplementary Table 13- Mann-Whitney test comparing the Roman population to all others in a dataset of British, Irish, Dutch and an Iron age and Anglo-Saxon individuals.

Population	P-value	Significance
Iron Age (M1489)	1.000	N.S.
Wales	0.697	N.S.
Anglo-Saxon (NO3423)	0.286	N.S.
North_West	0.208	N.S.
North	0.194	N.S.
Scotland	0.021	*
West_Midlands	0.013	*
South_West	0.009	**
South_East	0.007	**
East_Anglia	0.003	**
East_Midlands	0.003	**
Yorks_Humberside	0.003	**
Irish	0.000	***
dutch	0.000	***

Supplementary Table 14 - Imputed genotypes for each sample ancient at HIrisplex positions.

SNP	Ref	Alt	3DRIF-16	3DRIF-26	6DRIF-18	6DRIF-21	6DRIF-22	6DRIF-23	6DRIF-3	M1489	NO3423
rs11547464	G	A	GG	GG	GG	GG	GG	GG	GG	GG	GG
rs885479	G	A		GG	GG	GG	GG	GG	GG	GG	GG
rs1805008	C	T	CC	CC	CC	CC	CC	CC	CC	CC	CC
rs1805005	G	T	GG	GG			GC	GG	GG		GG
rs1805006	C	A	CC	CC	CC	CC	CC	CC	CC	CC	CC
rs1805007	C	T	CC	CC	CC	CC	CC	CC	CC	CC	CC
rs1805009	G	C	GG	GG	GG	GG	GG	GG	GG	GG	GG
rs2228479	G	A	GA	GG	GG	GG	GA	GG	GG	GA	GG
rs1110400	T	C	TT	TT	TT	TT	TT	TT	TT	TT	TT
rs28777	C	A	AA	CA	AA	CA	CA	AA	AA		AA
rs16891982	C	G	GG		GG	CG	CG	GG	GG		GG
rs12821256	T	C	TC	TT	TT	TT	TT	TT	TC	TT	
rs4959270	C	A	CC	CA	CC	CC	CC	AA	CC	CA	CA
rs12203592	C	T	CC	CC	CC	CT		CC		CC	CC
rs1042602	C	A	CC	CC	CC		CC	CC	CA	CA	CC
rs1800407	C	T	CT	CC	CC	CC	CC	CT		CT	CC
rs2402130	G	A	AA	GG	AA	AA	AA	AA	AA	GA	AA
rs12913832	A	G	AA	AA	GG		AG	AA	AG		GG
rs2378249	G	A	AA	AA	AA	AA	AA	GA	AA	AA	AA
rs12896399	G	T	TT	GG	GG	GT	GG	GG	GG	GT	TT
rs1393350	G	A	GG		GG	GG			GG		GA
rs683	C	A	CA	CC	AA	CA	CA	CC	CA		CA

Supplementary Table 15 - Phenotype prediction using according to the HIrisplex webtool.

SampleID	Eye phenotype	blue eye	intermediate eye	brown eye	Hair phenotype	blond hair	brown hair	red hair	black hair	light hair	dark hair
3DRIF-16	Brown	0.005	0.059	0.937	Black/Brown	0.273	0.478	0.002	0.247	0.53	0.47
3DRIF-26	Brown	0.000	0.014	0.985	Black/Brown	0.046	0.445	0.001	0.509	0.059	0.941
6DRIF-18	Blue	0.858	0.081	0.061	Blonde	0.729	0.233	0.003	0.035	0.953	0.047
6DRIF-21	N/A	0.000	0.000	0.000	Black/Brown	0.048	0.543	0.001	0.408	0.099	0.901
6DRIF-22	Brown	0.019	0.065	0.916	Black/Brown	0.125	0.337	0.001	0.537	0.173	0.827
6DRIF-23	Brown	0.001	0.041	0.958	Black/Brown	0.169	0.532	0.003	0.296	0.347	0.653
6DRIF-3	Brown	0.079	0.153	0.768	Blonde/Brown	0.489	0.447	0.003	0.061	0.858	0.142
Iron Age (M1489)	N/A	0.000	0.000	0.000	Blonde/Brown	0.418	0.389	0.005	0.188	0	0
Anglo-Saxon (NO3423)	Blue	0.963	0.027	0.010	Blonde	0.748	0.213	0.004	0.035	0.959	0.041

Supplementary Table 16 - Imputed genotypes at the SCL24A5, SLC45A2, TYRP and LCT loci.

	SCL24A5 rs1426654		SLC45A2 rs16891982		TYRP1 rs2733831		HERC2 rs12913832		LCT rs4988235	
**Sample**	**Allele 1**	**Allele 2**	**Allele 1**	**Allele 2**	**Allele 1**	**Allele 2**	**Allele 1**	**Allele 2**	**Allele 1**	**Allele 2**
Iron Age (M1489)										
3DRIF-16										
3DRIF-26										
6DRIF-18										
6DRIF-21										
6DRIF-22										
6DRIF-23										
6DRIF-3										
Anglo-Saxon (NO3423)										

Supplementary Table 17 - Imputed genotypes at SNPs associated with blood groups.

Sample	rs8176719	rs8176746	rs8176747	blood group
3DRIF-16	-/-	G/G	C/C	O
3DRIF-26	-/-	G/G	C/C	O
6DRIF-18	-/-	G/G	C/C	O
6DRIF-21	-/-	G/G	C/C	O
6DRIF-22	C/C	G/G	C/C	A
6DRIF-23	-/-	G/G	C/C	O
6DRIF-3	-/-	G/G	C/C	O
Iron age (M1489)	-/-	G/G	C/C	O
Anglo-Saxon (NO3423)	-/C	G/T	C/G	A or B

Supplementary Table 18 – Number of SNPs called in ancient samples.

Sample	Number of SNPs Hellenthal et al. (2014)	WTCCC1/Irish/Dutch
3DRIF-16	248355	136335
3DRIF-26	336069	182143
6DRIF-18	328974	179979
6DRIF-21	342010	186920
6DRIF-22	338210	184940
6DRIF-23	243126	132510
6DRIF-3	399115	218462
Iron Age	211233	113687
Anglo Saxon	324454	177175

Supplementary Note 1

1. Archaeological sites

1.1 Driffield Terrace

York (Eboracum) in the North of England, founded around AD 71, was an important military base and major urban settlement, which became the northernmost of the Roman provincial capitals in the early third century AD. Despite its relatively peripheral location, archaeological and historical evidence attests the city's extensive connections throughout the Empire through the movement of people and goods [31]. As typical for the Roman period, the cemeteries of York were located outside the settlement area, lining the roads leading away from the town (Supplementary Fig. 1). Two excavations less than 50m apart conducted by York Archaeological Trust in 2004 and 2005 in the area of The Mount, an elevation ~500m southwest of the civilian settlement apparently the location of high-status burials, revealed a previously undisturbed part of the cemetery . In total, 82 skeletons were recovered from 1–3 and 6 Driffield Terrace [32,33]. The burials followed no specific alignment and there was some intercutting of graves, indicating that the burials had occurred over a period of time (Supplementary Fig. 1). This conclusion was supported by dating evidence dividing the burials into several phases from the late 1st/early 2nd to at least the late 3rd, possibly continuing into the 4th century AD. Overall, 70.8% of those individuals where either osteological or contextual evidence for decapitation could be observed had been decapitated[34] (46/65).

Most individuals were adults (75/82, 92.6%), 66 (88.0%) of whom were males [34]. The majority of the males were young middle adults (26-35 years), while the remainder were old middle adults (36-45 years) or young adults (18-25 years; see Supplementary Table 1). One young middle adult female was present (Skeleton 3DRIF-42). Eight adults were unsexed so males made up 98.5% of the sexed adults (66/67). Seven individuals were non-adults (8.5%). These included three older adolescents (aged ~16-19 years of age) who were likely socially equivalent to the young adults. The remaining non-adults comprised a foetus (~30-32 weeks in utero), a neonate (0-1 month), a young juvenile (1-2 years) and an older juvenile (6-7 years). Analysis of the disarticulated remains suggested an infant of ~1-3 months may also have been buried at the site.

Evidence for trauma was prevalent. This included healed cranial trauma (23.3% of 60 individuals, most frequently affecting the left side of the vault), and dental fractures (32.2% of 59 adults; 3.1% of 1590 teeth). Injuries to the skull are most often interpreted as inter-personal violence, and the typically higher frequencies of cranial injuries on the left side has usually been inferred to indicate hand-to-hand combat with a right-handed assailant [35]. However, accidents can also lead to cranial trauma (ibid.). At Ephesus, 44% of the healed cranial trauma was attributed to blunt force injuries [36], but the majority of lesions were different in character to those at Driffield Terrace.

Ante-mortem fractures were also observed in the limbs and torso: 20 individuals had fractured limb bones, and 18 had fractured their vertebrae or ribs. The limb bone most frequently fractured was the first metacarpal (5.3%), followed by the third metacarpal (avulsion fractures, 2.8%), and fibula (2.8%). Fractures of the base of the first metacarpal occur most frequently in the dominant hand of male individuals, and are typically sustained through punching a firm surface with a closed fist, so tend to

occur during fights or sporting activities [37,38]. One individual had suffered a fracture to the right scapula blade that was still healing at the time of death. Scapular blade fractures are usually caused by a direct blow [39] and may be indicative of interpersonal violence [35,40]. One individual had a probable healed blade injury to the distal third of the right femur.

Peri-mortem sharp and blunt force trauma was also observed. A young adult male had a peri-mortem butterfly fracture to the midshaft of his right ulna [originally described by 41], which may indicate a parry fracture [42]. Another young middle adult male individual had a series of small depressions on both sides of the pelvis, focused on the iliac crest and anterior superior iliac spines, identified by Tucker [41] as carnivore bite marks. Forty individuals (all adult males) had peri-mortem cuts to the neck, but in one of these the injury was possibly a stab wound rather than connected to decapitation. The frequency of cuts to the spine was highest among the young middle adults, followed by old middle adults.

Cribra orbitalia and dental enamel hypoplasia (DEH) indicate episodes of childhood stress, potentially involving poor diet, infectious disease, and/or parasite loads [43]. The prevalence of cribra orbitalia at Driffield Terrace was higher than average for the Roman period, both in terms of the number of orbits affected (24.6%) and the number of individuals affected (30.6%). Roberts and Cox [44] reported the former to be 16.9% and the latter to be 9.6% on average for Romano-British populations. The proportion of teeth with DEH at Driffield Terrace (17.9%) was nearly double the Romano-British average [9.1% 44], but lower than the frequency of DEH seen at Mill Mount, York [45]. However, adult stature at Driffield Terrace was not reduced. The mean male stature was 170.7cm (based on using the 'black' formula for individuals of African and Mixed ancestry, and the 'white' formula for individuals of Caucasian and unknown ancestry), which is slightly higher than the mean male stature of 169 cm reported for Roman Britain [44]. The female individual was slightly taller than average for the Roman period [159 cm 44], at 160.9cm tall.

Periosteal reactions indicative of inflammation or infection [42] were frequent, particularly in the lower limbs. Evidence for respiratory infections was also observed, including maxillary sinusitis, and new bone formation on the visceral surfaces of the ribs. Frequencies of dental disease and joint disease were low, no doubt reflecting the young age bias of the population.

A thorough presentation of the palaeopathological data pertaining to Driffield Terrace can be found in the full skeletal report [34].

1.2 Melton

A complex series of settlements and burials dating from the Bronze Age to the medieval period were discovered in 2004-2005 to the east of the village of Melton, East Yorkshire, at the southern edge of the Yorkshire Wolds 1.5km north of the Humber Estuary [46]. Prior to the first millennium BC, burial evidence comprised a round barrow dating to the third or second millennium BC that lacked an identified central burial, and cremation burials (one of which was contained in an urn) from the second millennium BC. A small Iron Age linear cemetery contained inhumation burials dating to the sixth to fifth centuries BC, and two square barrows dated to the second half of the first millennium BC (*ibid.*). In the first century BC/ early first century AD, human and animal inhumation burials had been interspersed amongst the abandoned buildings of a late Iron Age ladder settlement (*ibid.*, p374). These human remains predominantly consisted of adult females and non-adults. It was one of these individuals M1489), an

adult female aged 26-35 years buried in a flexed position in a north-south orientation, which was sampled for this project. This individual was radiocarbon dated to 210 BC to 40 AD, and was considered most likely to date to the first century BC (*ibid*.) Later, five seventh century burials had been located nearby at the junction of two tracks (*ibid.*).

1.3 Norton on Tees

Two Anglo-Saxon cemeteries were excavated in the village of Norton, Teesside, north east England. They were located 200m apart and one of these, Norton East Mill, dated to the pagan Anglo-Saxon period (550-650AD), while the adjacent cemetery, Norton Bishopsmill, dates to 650-910AD and was Christian. The pagan cemetery contained 120 burials, many of which were furnished with grave goods, while the Christian burial ground contained the unfurnished burials of 100 skeletons. Analysis of the skeletal remains has revealed differences in the age and sex distributions as well as the pathology at the two sites [47,48].

Osteological and palaeopathological data for the skeletons sampled for this project can be found in Supplementary Table 2.

Supplementary Note 2

1. Isotope analysis

1.1 Methods

Multi-isotope data for six of the seven individuals has been previously published [6,49]. A molar tooth was submitted for isotope analysis for the remaining skeleton 26 from 3 Driffield Terrace (3DRIF-26) and collagen extraction for carbon and nitrogen isotope analysis was re-attempted on long bones of individuals where rib samples had previously failed to yield a viable product (6DRIF-3, 6DRIF-21, 6DRIF-22, see reference [50]. For 6DRIF-18, two sections of tooth root remaining from the original sample were processed.

1.1.1 Sr and O isotope analysis.

The enamel of 3DRIF-26 was mechanically cleaned with clean tungsten carbide dental burrs to a depth of c. 100 µm and all adhering dentine removed following the procedure detailed in reference [51]. Regions of carious, opaque, cracked or discoloured enamel were avoided. Samples were removed from the tooth and transferred in sealed containers to the class 100 HEPA-filtered laboratory facility at the NERC Isotope Geosciences Laboratory, Keyworth, UK and to the Stable Isotope Laboratory, University of Bradford, UK for strontium and oxygen isotope analysis respectively. Dentine samples for carbon and nitrogen isotopes were removed from the remaining tooth root.

In the clean laboratory, samples for strontium were cleaned in high purity acetone, washed ultrasonically in water (Millipore Alpha Q, <1 ppb total heavy metal content) to remove adhering particulates and leached on a hot plate for two hours following the established laboratory procedure. Samples were weighed and spiked with an ^{84}Sr tracer before being dissolved in Teflon-distilled 16 M HNO_3. Strontium was collected using Dowex© resin ion-exchange columns. Strontium isotope ratio and concentration were determined by Thermal Ionisation Mass Spectrometry (TIMS) using a Thermo Triton multi-collector mass spectrometer. The prepared samples were loaded onto a single outgassed Re filament with TaF following the method of Birck (1986). ^{87}Sr/^{86}Sr ratios were normalised to a value of 0.710250 for the international standard NBS987. External reproducibility was estimated at ± 0.003 % (2σ). Laboratory contamination monitored by within-run laboratory blanks was negligible (~ 100 pg).

For oxygen and carbon isotope determination of structural carbonate powdered enamel samples were treated with 1.7% NaOCl solution for 30 min to remove organic matter and rinsed with distilled water, before adding 0.1M acetic acid for 10 min to remove exogenous carbonate following a protocol modified after Sponheimer (1999). Rinsed and freeze-dried samples were weighed in duplicate and isotopic compositions were measured using a Finnigan Gasbench II connected directly to a Thermo Delta V Advantage continuous flow isotope ratio mass spectrometer. Enamel carbonate was reacted with anhydrous phosphoric acid at 70°C to release CO_2 gas from which $\delta^{18}O_{VSMOW}$ and $\delta^{13}C_{VPDB}$ were determined using a CO_2 reference gas. Data were normalized by means of a linear calibration equation derived from a plot of accepted versus measured values for two internal standards, Merck Suprapur

$CaCO_3$ and OES (ostrich egg shell), and the NBS19 international standard. Analytical precision was ± 0.1 ‰ (1 SD) for carbon isotope ratios and ± 0.2 ‰ (1 SD) for oxygen isotope ratios determined using an internal enamel laboratory standard. The normalized carbonate $\delta^{18}O$ values were converted to phosphate values using equations by Chenery et al. [52] and Iacumin et al. [10] and then to drinking water values using equation 6 from Daux et al. [53] (see Supplementary Table 3).

1.1.2 Carbon and nitrogen isotope analysis of collagen.

Bone and dentine collagen was extracted according to the Longin [54] method, modified according to the recommendations by Collins and Galley [55]. The surfaces of all bone and tooth samples were abraded with the aid of a drill. Samples of approximately 200mg (bone) and 30mg (dentine) were demineralized in 0.5 M HCl at ~4°C over several days, then rinsed to neutrality with Milli-Q® ultrapure water. The resulting 'collagen ghosts' were gelatinized over 48h in a pH 3 HCl solution at 70°C, after which insoluble residues were removed with a 50-80μm Ezee® filter (Elkay). The solution was freeze-dried over 48h and the resulting product weighed in order to determine collagen yield. Aliquots of ~1mg were then weighed in duplicates into ultra-clean tin capsules. $\delta^{13}C_{(‰VPDB)}$ and $\delta^{15}N_{(‰AIR)}$ were determined on a Sercon 20-20 continuous-flow isotope ratio mass-spectrometer using internal standards which are regularly calibrated against internationally certified reference materials (IAEA-CH-6, -CH-7, -N-1, -N-2 and USGS-40). Results were normalized by a two-point calibration of measured versus expected values. Analytical precision determined by repeat analysis of an internal collagen standard was ±0.1‰ (1 SD) for both carbon and nitrogen isotope ratios. Collagen quality was assessed using criteria by DeNiro [56] and Van Klinken [57]. While all samples yielded sufficient product for analysis, results for 6DRIF-21 (bone sample) were excluded on grounds of poor preservation. Results are presented in Supplementary Table 4.

1.2 Results

1.2.1 Strontium

Six of the genotyped individuals from Driffield Terrace have strontium isotope compositions consistent with humans in Britain (Supplementary Fig. 2) and with biospheres hosted by the sedimentary silicate rocks of north eastern England, in or close to the Vale of York [2,see 6,58].. In contrast, 3DRIF-26 has an extremely unusual strontium profile for Britain having a low ratio of 0.70777 coupled with a strontium concentration of 177 mg/kg, the highest from any burial at Driffield Terrace [6,49].. This ratio is inconsistent with the majority of rock types in England where only sedimentary marine limestones, and igneous basalts and mantle rocks such as ophiolites, have been found to host biospheres below 0.7080 [58]. However, 0.70777 is extremely low for humans living on marine carbonate terrains in Britain; although many limestones themselves have $^{87}Sr/^{86}Sr$ values as low as 0.7068 [59], in a temperate, maritime environment plants growing on them rarely do [58,60]. This is due to a combination of silicate inclusions, i.e. 'dirty' limestones, and the addition of atmospheric strontium in the form of rainwater to the biosphere strontium pool. These mechanisms raise the strontium isotope ratios of the plants and consequently also the humans [61,62]. As a result, humans excavated from chalk and limestone regions in Britain typically cluster at the bottom left in Supplementary Fig. 3, exhibiting $^{87}Sr/^{86}Sr > 0.7078$, coupled with strontium concentrations < 120 mg/kg. For example, the lowest human $^{87}Sr/^{86}Sr$ in Supplementary Fig. 3 is 0.70784 with 38 mg/kg. Similarly, at the Middle Iron Age cemetery of Wetwang located on the Chalk of the

Yorkshire Wolds, one of the purest marine limestones in Britain, the mean concentration was 54 +/- 16 mg/kg (1 SD, n = 34) and the least radiogenic $^{87}Sr/^{86}Sr$ of 0.70798 was coupled with a concentration of 26 mg/kg [62].

If origins on limestones in temperate maritime regions of Britain are therefore unlikely places of origin, this leaves only igneous basalts and ophiolites that can provide strontium ratios below 0.7080 in British humans. There are restricted outcrops of Precambrian and Palaeozoic basalts in Scotland and Wales and ophiolites in the Lizard peninsular in the extreme southwest of England which could provide the low value seen in 3DRIF-26. Tertiary dyke swarms such as the Cleveland Dyke, occur across northern Britain. In Northern England, however, these do not constitute significant sources of agricultural land and it is therefore not expected that the $^{87}Sr/^{86}Sr$ of humans in these regions would approach their low values. The major source of such rocks is the Tertiary Volcanic Province which is primarily found down the western seaboard of Britain and in the north of Ireland. For example, the individual immediately to the left of 3DRIF-26 in Supplementary Fig. 3 is a Viking period skeleton from the Outer Hebrides in Scotland which lies within the Tertiary Volcanic Province [61].

1.2.2 Oxygen

Five of the seven individuals from Driffield Terrace have phosphate oxygen isotope ratios ($\delta^{18}O_p$) that fall within the 2 SD range of archaeological humans from Britain [4], as indeed do most of the individuals from Roman York (Supplementary Fig. 2). This range is wider and probably more robust than the one used in [6], being based on significantly more samples (n=615) than were available previously (n=42, see [1]. It can be regarded as the current best estimate for the range of $\delta^{18}O_p$ values expected for individuals who grew up in Britain; however, it is still open to revisions as more samples become available.

Individuals who plot to the right of the estimated British range have higher $\delta^{18}O$ values that indicate the ingestion of rainwater falling in warmer, drier climates at lower latitude, that is relatively enriched in ^{18}O [63]. However, higher $\delta^{18}O$ values can also result from localised environmental or cultural processes such as evaporation of drinking water during storage in lakes or man-made containers [64], or the consumption of processed fluids such as milk, beer, wine and boiled foods or beverages [53,65,66]. Two individuals fall outside the 2 SD range for Britain: 6DRIF-21 and 3DRIF-26.The $\delta^{18}O_p$ value for 6DRIF-21 of 19.8‰ was, prior to this study, the most ^{18}O-enriched value reported from Roman Britain, although oxygen isotope ratios >19‰ have been observed in a small number of samples, particularly those from Southern and Western Britain [1,3,67,68]. While some of these may have been migrants, there is a general consensus now that at least the definition of the upper boundary of the British range is very much in flux, given that the known mechanisms for the modification of local rainwater into drinking water almost exclusively involve ^{18}O-enrichment and not depletion [see 69]. The results of the genetic analysis, combined with 6DRIF-21's enamel lead isotope ratios which are strongly suggestive of childhood exposure to anthropogenic lead from English mines (but are not necessarily conclusive by themselves since British lead was widely exported in the Roman period, see [70], demonstrate that even $\delta^{18}O_p$ values approaching 20‰ are probably not incompatible with a British origin. The $\delta^{18}O_p$ value of 21.7 ‰ for 3DRIF-26 (calculated from the measured carbonate value using Iacumin et al.[10] is far higher than any value that would be expected from an inhabitant of Britain, falling well outside the +4 SD range of the complete

Evans et al. (2012) data-set (n=677). Taken together with the unusual strontium profile, it appears highly likely that this individual was an immigrant to Britain from a warmer climate.

1.2.3 Carbon and Nitrogen Stable Isotope Results

$\delta^{13}C$ and $\delta^{15}N$ values of five of the genotyped individuals fall within or marginally outside (6DRIF-3) ±2 SD of the mean for Romano-British humans from York (n=173) (Supplementary Fig. 4). These data are consistent with the local Romano-British diet which was very predominantly based on terrestrial C_3-resources, but probably with small contributions of marine protein to the diet of at least parts of the population [7,8]. Two of the individuals (3DRIF-26 and 6DRIF-18) have dentine $\delta^{15}N$ values which are higher than the York mean +3 SD (or further than 1.5 interquartile ranges from the median). Consequently, neither individual was consuming the typical York diet, at the time when the sampled tooth roots were forming, between c. 7 and 14 years of age[71]. Indeed, the two data-points are equally unusual when compared with the sizeable human data-set available from all of Roman Britain (mean $\delta^{13}C$ -19.6 ± 0.7 and $\delta^{15}N$ 10.2 ± 1.3‰ (1 SD)). Only 8 individuals in the entire data-set (n=858) have $\delta^{15}N$ values ≥13‰ [1,6,7,23,72–77]. Dietary variation between individuals are often the result of social or economic differentiation; however, with differences as stark as presented here, migration from areas with very different subsistence regimes or environmental baselines must be considered as an alternative explanation [see 6,78]. This is almost certainly the case for 3DRIF-26 where a shift of 2.4‰ between the $\delta^{15}N$ value in late childhood/adolescence (root dentine) and adulthood (rib) is consistent with dietary change on migration to York or places with a similar dietary regime (Supplementary Fig. 4). Combined with his extremely high $\delta^{18}O$ value, an origin in an environment where aridity effected significant ^{15}N-enrichment in soils and plants seems the most likely explanation [79–81]. 6DRIF-18 has been discussed in detail elsewhere [see 6]. The results of the genetic analysis which indicate British ancestry, provide an important new dimension to the interpretation and emphasise that dietary variation within the British Isles in the first centuries AD is not yet fully explored.

1.3 Discussion - The Geographical Origin of 3DRIF-26

Moving south and east through the Roman Empire, volcanic basalts and lavas and mantle rocks such as ophiolites which can provide low $^{87}Sr/^{86}Sr$ values to soils and plants occur at the surface with increasing regularity. In southern Europe, volcanic rocks occur in western Italy, Sardinia and central Spain and in northern Europe there are extensive volcanic terrains in the European Cenozoic Volcanic Province which encompasses the Central Massif in France, the High Eifel, Westerwald, Vogelsberg, Rhön in Germany, Lower Silesia in Poland, the Eger Graben in the Czech Republic and down through the inner eastern and western Carpathians [82,83] . Localised outcrops of volcanic lavas and the ophiolites of the Dinaric-Hellenic belt are found down through the Balkans and Greece and in Cyprus and Turkey [84,85]. Volcanic lavas and ophiolites are found extensively throughout Turkey and volcanic rocks in the Near East south of the Caucasus and across to the Caspian Sea. They continue down through Iran, Syria, Lebanon, Israel and Jordan and Saudi Arabia and are found further south in Sudan and Ethiopia. To the west around the southern Mediterranean, there are also significant outcrops in Libya ([84,85]. Recently, [86] undertook strontium isotope biosphere mapping in northern Israel around the Sea of Galilee and their mean values

range from 0.70465 to 0.70687 for basalts and up to 0.7090 for calcareous sandstones. An averaged value for the region of 0.7075 was proposed by [87].

The high oxygen isotope ratio of 3DRIF-26 equates to a rainwater $\delta^{18}O$ value of -0.3 ‰ using the carbonate-to-phosphate conversion equation by Iacumin et al. [10] (see Supplementary Table 3). Assuming a typical error of ±2.5‰ for [53] phosphate-to-drinking water Equation 6 [76] and therefore drinking water values between -2.8‰ and +2.5‰, it appears that a childhood in Europe is unlikely since, here, the mean annual $\delta^{18}O$ of modern rainfall rarely exceeds -3 ‰ [88]; IAEA/WMO 2015). Although we cannot rule out the possibility that weather patterns may have changed and higher temperatures and thus higher $\delta^{18}O$ in precipitation and drinking water existed locally, palaeoclimatic records indicate a broad trend towards a cooler, drier climate from the mid- 3rd century CE, amid a general setting of climatic instability [89,90].

Turning to the Levant, [91] citing amongst others [92] study of rainfall in the Upper Jordan Valley, estimated an upper limit of only -4.5 ‰ for most of the Levantine coast, with values reducing further, east of the Levantine coastal plain, suggesting none of these regions would provide a place of origin for 3DRIF-26. Similarly, [93] who conducted a survey of water $\delta^{18}O$ data in modern day Israel and Jordan reported values above -3‰ only for individual coastal aquifers in the south, with the majority of values in the region well below -4‰. Mean annual rainwater $\delta^{18}O$ values above -3 ‰ are found on the edges of the Roman Empire, such as in parts of the North African coast, specifically in Libya, western Egypt and further east into Saudi Arabia and Iran [88,94] . Nevertheless, as previously discussed, $\delta^{18}O$ values of the water actually drunk by people can be increased during evaporation by wind or heat, and heating and other cultural modifications of drinking water (e.g. brewing). In hot climates, therefore, evaporation of drinking water sources which are not regularly replenished by rainwater would produce higher $\delta^{18}O$ values in humans drinking them and this has been documented empirically. For example, from a longitudinal study of Egyptian mummies dating from ~ 6000 to 1310 BP, [95] concluded that increasing aridity over this period was raising the $\delta^{18}O$ of Nile water from -1.6 to +1.5 ‰ and by the Greco-Roman Period human phosphate oxygen isotope ratios ranged between 20.5 to 24.5 ‰. Similarly, in a seminal study in Egypt's Nile Valley, Iacumin et al. (1996) measured $\delta^{18}O$ in both enamel carbonate and phosphate and obtained virtually the same range for humans of 30.2 to 32.5 ‰ and 20.6 to 24.5 ‰ respectively in which 3DRIF-26 comfortably sits. In burials from Nubia, mean enamel phosphate $\delta^{18}O$ values of 24.7 ‰ were reported [13] which is considerably higher than 3DRIF-26 but suggests the oxygen isotope ratios of ancient humans may be higher than modern precipitation maps would predict.

Critically, in arid regions, low rainfall would also bring limestone terrains within the strontium isotope range of 3DRIF-26 due to the reduced contribution of rainwater strontium to plants. Such limestone rocks are found across large regions of Libya, Egypt, Sudan and the Levant and, in contrast to many volcanic basalts, they frequently host fertile agricultural land. The mean value for $^{87}Sr/^{86}Sr$ in Theban limestones and Nile sediments is reported to be 0.70777 [59,95], a value identical to that of 3DRIF-26. A $^{87}Sr/^{86}Sr$ range of 0.7075 to 0.7083 was also determined at archaeological sites on the limestone of western Jordan [93]. Similarly, [96] studied archaeological humans from Egypt and northern Sudan and although none of the sites were located in volcanic regions, they estimated the local $^{87}Sr/^{86}Sr$ range to be 0.7073 to 0.7079. The mean $\delta^{18}O$ for human enamel carbonate here was 31.4 ‰ with a range of 29.2 to 35.3 ‰ whilst the mean value for $\delta^{13}C_{carb}$ was -11.8 ‰ with a range of -13.4 to -7.2 ‰; 3DRIF-26 therefore falls within their identified 'local range' for strontium, oxygen and carbon isotopes. Carbon and nitrogen stable isotope

values for 3DRIF-26's root dentine, in combination with his high $\delta^{18}O$, suggest origins in an area where aridity had raised the $\delta^{15}N$ of soils and plants but where neither marine resources nor C_4-plant-derived protein made a significant contribution to the human food chain. Supplementary Fig. 5 shows 3DRIF-26 in comparison with mean carbon and nitrogen isotope values (±2 SD) for mostly Roman-period populations in Europe, North Africa and the Near East. It is apparent that 3DRIF-26's dietary profile is not easily consistent with available data for European Mediterranean or Near Eastern populations. This impression would not change significantly, if earlier or later samples from the same areas were included, although it is a serious limitation that there are currently few available human data from Near Eastern desert regions, where bone preservation is usually poor. Nevertheless, exceptionally dry places may possibly also be ruled out, as comparison with isotope data from the Dakhleh Oasis in Egypt suggests: here, human $\delta^{15}N$ values are generally much higher, averaging around 18‰ [97,98]. The best fit for 3DRIF-26's carbon and nitrogen isotope data are therefore currently human populations from the Lower Nile Valley in Egypt [10], possibly also from Nubia, although a greater C_4-component in diet of most individuals and relatively low $\delta^{15}N$ in human groups that best fit the time-frame [13] makes the latter appear less likely. While the Lower Egyptian data are not Roman in date but from the 4th century BCE or earlier, the isotopic record for diet in this area is remarkably constant over several millennia it may not be unreasonable to assume that it had not significantly changed by the 3rd-4th century CE (but see [95]). The fact that multiple isotopic proxies (Sr, O, C, N) for 3DRIF-26 appear consistent with the same general area, Lower Egypt, should not be misunderstood as a secure assignation of origin, however, especially given that reference values used are not from the Roman period and comparative data from other regions (such as arid parts of the Levant or Eastern Syria) that would provide a good fit on theoretical grounds, are not currently available. The similarities should therefore rather be taken as evidence for the kind of environment in which 3DRIF-26 spent his childhood than as a positive identification of the area of origin.

1.4 Conclusions

Sr-isotope and concentration and oxygen isotope data for 3DRIF-26 is inconsistent with Europe and consistent with origins on basalt or limestone terrains in a hot, dry region of northern Africa or the Levant. Dietary (C and N) isotope data are consistent with this and suggest a childhood in an area where aridity had a significant effect on $\delta^{15}N$ values but were C4-plants or marine resources made no significant contribution to the protein intake. Origins for all other individuals in Britain cannot be ruled out but have potentially important implications for the definition of the British isotope range.

2. Ancient DNA analysis

2.1 Sample preparation

2.1.1 DNA sampling and extractions

Ancient DNA sample processing was done at the Ancient DNA lab, Smurfit Institute, Trinity College Dublin (Ireland), in clean-room facilities exclusively dedicated for this purpose. We analyzed 7 samples from Driffield Terrace, 1 Iron Age (M1489) from Melton and 1 Anglo-Saxon (NO3423) from Norton on Tees. The surface of bone samples was decontaminated by UV light exposure for 10 minutes each side and cleaned with a drill bit. The densest part of the petrous bone [99] was excised using a circular saw.

Extractions of approximately 150 mg of bone powder were done with a silica-column-based method as previously described [100] and modified [101]. Undigested pellets were subsequently re-extracted, purified with Minelute columns (Qiagen MinElute PCR Purification Kit, Qiagen, Hilden, Germany) and eluted in 50 ul of dH2O. Blank controls were included throughout extractions, library preparation and PCR reactions to monitor the possibility of contamination (Supplementary Table 8).

2.1.2 Library preparation and amplification

Next-generation sequencing libraries were constructed from extracted DNA using the method described in [102] with modifications [99,103]). Briefly, we used T4 DNA polymerase buffer (Thermo Scientific) instead of Tango buffer in the Blunt-End Repair step; replaced Solid Phase Reversible Immobilization (SPRI) purification with Minelute Purification, and heat inactivated Bst Polymerase by incubating the libraries for 20 minutes at 80°C instead of an extra purification step. Indexing PCRs (12 to 14 cycles) were done in a room where only aDNA is amplified, using AccuPrime Pfx Polymerase (Invitrogen) and a different indexing oligo for each sample. PCR reactions were quantified using Quant-iT dsDNA HS Assay kit (Invitrogen, Oregon, USA) and with Agilent 2100 Bioanalyzer High Sensitivity DNA kit and pooled equimolarly.

2.1.3 Illumina Sequencing

NGS libraries were sequenced in an Illumina MiSeq (50 cycle kit, single-end reads mode, Institute of Molecular Medicine (IMM), Trinity College Dublin) for endogenous DNA screening, along with PhiX control at 1%. Samples with endogenous DNA content above >20% were prioritized for sequencing to higher coverage. We then amplified the same libraries with 3-4 distinct indexing oligos for each sample in order to increase index diversity in each lane. PCR products were then pooled and sequenced to approximately 1X (5.5 lanes) in a Illumina HiSeq 2000 (100 cycle kit, single-end reads mode; Macrogen).

2.2 Next-Generation Sequencing Reads Processing and Filtration

2.2.1 NGS reads processing, alignment and filtration

Next-generation sequencing reads were trimmed with Cutadapt v. 1.3 [104], discarding reads shorter than 25 bp, and allowing for a minimum overlap of 1 bp between the read and the adapter (adapter sequence is 'AGATCGGAAGAGCACACGTCTGAACTCCAGTCAC'). Two bases from each side of reads were removed with setk (https://github.com/lh3/seqtk). The Burrows-Wheeler Aligner v.0.7.5a-r405 (BWA) [105] was used to map sequencing reads to the human reference genome (UCSC hg19), filtering by base quality 15 and disabling seed length as recommended for aDNA data [106]. SAMtools v.0.1.19-44428cd [107] was used to exclude PCR duplicates and reads with mapping quality inferior to 30. Genome depth of coverage was estimated using depth-cover (https://github.com/jalvz/depth-cover). Results are shown in Table 1 in the main text.

2.2.2 Preparation of genotype data of present-day populations

2.2.2.1 Hellenthal et al. (2014) genotype data

We made use of a publicly available dataset used to investigate human admixture through historical times [108]. Autosomal SNP positions were mapped to b37 (GRCh37) of the human genome and PLINK [109] was used to filter by genotyping rate (SNP genotypes absent in > 10% of individuals were removed) and minor allele frequency (MAF) of 0.5%. We then selected individuals of European, Middle Eastern, West Asian and North African ancestry for subsequent analysis, resulting in a dataset comprising of 780 individuals and 468.538 SNPs. To account for the relatively low genome coverage of our ancient samples, we converted the present-day individuals genotypes to homozygous at random [110].

2.2.2.2 Irish, British and Dutch genotype data

In order to infer the ancient samples' affinity with northwestern Europeans, we combined 3 publicly available genotype datasets: British (n=2277)[111], Irish (n=399)[112] and southern Dutch samples from the North Brabant, Limburg and Zeeland regions (n=399)[113]. These datasets were independently filtered by the following criteria: we removed WTCCC-recommended individuals and SNPs, removed A/T and G/C SNPs, remapped to GRCh37 reference coordinates and forward strand, excluded SNPs in Hardy-Weinberg disequilibrium or with > 2% missingness, removed individuals with overall heterozygosity deviating from the cohort median and removed individuals whose recorded sex did not match sex determined by X-chromosome heterozygosity. Additionally, we excluded individuals from London (known to be cosmopolitan) and individuals from island populations due to very low sample size. After filtering and merging, the final dataset is composed of 3075 individuals, with 256712 SNPs in the intersection.

2.2.3 Variant calling in ancient individuals

Base quality scores at the end of reads were recalibrated with mapDamage v.2.0 [114] to exclude potential deamination residues from subsequent analysis. We used the Genome Analysis Toolkit v.2.5 (GATK) [115], Pileup mode, to identify alleles present in ancient samples at SNP positions genotyped on present-day human datasets, keeping bases with quality superior to 30. Due to low genomic coverage (~1X), only one allele was selected at random for each SNP position and converted to homozygous [110]. These base calls were then converted to PLINK format [109]. Number of SNPs called in ancient for each analysis in the main paper is shown on Supplementary Table 18.

2.3 Sex determination of ancient samples

In order to determine the sex of the ancient samples analysed in the present study, we used a published software [116] which takes into account the ratio of X to Y chromosome reads. All samples from Driffield Terrace and the Anglo-Saxon individual were determined to be male and the Iron Age samples was identified as a female (Supplementary Fig. 7).

2.4 Contamination estimates and verification of authenticity

We followed two approaches for estimating contamination in the 9 ancient samples sequenced in the present study: 1) mtDNA and 2) X-chromosome contamination in males.

2.4.1 Estimation of Deamination rates

A subset of 1 million reads, untrimmed with *seqtk*, were analysed with mapDamage 2.0 [114] with the purpose of investigating deamination patterns (Supplementary Fig. 6). The values of cytosine deamination were determined to be between ~10-16% and are therefore consistent with the presence of authentic ancient DNA.

2.4.2 Mitochondrial DNA contamination estimates

Next-Generation Sequencing reads were aligned to the revised Cambridge Reference Sequence (rCRS; NC_012920.1) [117], using BWA filtering for base ($q \geq 30$) and mapping ($q \geq 30$) quality and duplicate reads were removed. We then used SAMtools [107] to obtain the mtDNA consensus for each ancient sample, which was then uploaded to HaploFind (https://haplofind.unibo.it; [118]), where mitochondrial haplogroup defining positions were obtained. Next, we did base calling for each position with SAMtools mpileup. We applied an identical approach as [99], in which contamination can be estimated by calculating the average proportion of mismatches in relation to the consensus sequence in haplotype defining positions. This resulted in low mtDNA contamination estimates in the range of 1.82±0.47%. If potential deamination changes were excluded by processing NGS reads with mapDamage 2.0 [114], a lower value of mtDNA contamination 1.32±0.48% was observed (Supplementary Table 5).

2.4.3 Estimation of contamination rates based on the X-chromosome

Since 8 individuals sequenced were determined to be males, we used ANGSD [119] to estimate X-chromosome contamination in these samples based on a previously published method [120]. In male individuals, which are haploid for the X-chromosome, mismatches at polymorphic sites can be interpreted as either contamination or sequencing errors. In order to account for the latter, polymorphic sites and also their adjacent bases are screened for mismatches, because sequencing errors should be observed at identical frequencies across the genome.

The present analysis was done by using default parameters and by using the provided mapFile ("RES/chrX.unique.gz") and hapFile ("RES/HapMapChrX.gz"). We only considered bases with base quality ≥ 20 and mapping quality ≥30. These restricted the analysis to unique regions of the X-chromosome and to HapMap X-chromosome polymorphisms.

Two methods were applied as in [120] using ANGSD. Briefly, in Method 1, the major and minor base are detected for each SNP position and adjacent sites, using the later to assess background error rates for each sample. While Method 1 assumes that errors between reads and sites are independent, Method 2 randomly samples a single read at each site prior to determining the number of major and minor bases. Contingency tables for both methods and contamination estimates are shown in Supplementary Table 6-7.

2.4.4 PMDtools

We used PMDtools [121] to select reads with evidence of deamination (PMD score 3) and compared sex determination (Supplementary Fig. 7) and Principal Component Analysis produced based on deaminated and unfiltered reads (Supplementary Fig. 8). This allowed rejecting the possibility of substantial modern human contamination affecting our results.

2.5 Principal Component Analysis

2.5.1 Procrustes transformation

For determining the affinity of the ancient samples to present-day populations, we used, at a first instance, a worldwide dataset [108] from which we selected 780 individuals of European, Middle Eastern, West Asian and North African populations and used PLINK v.1.07 [109] and PLINK v.1.9 [122] to merge individual aDNA samples with publicly available genotype data, excluding all problematic SNPs in the ancient samples, SNPs with Minimum Allele Frequency (MAF) < 0.05 and genotyping rate < 0.1. We then used smartpca [123,124] to perform Principal Component Analysis, excluding SNPs in Linkage Disequilibrium (r2 > 0.2). The R [125] package "vegan" (http://cran.r-project.org/web/packages/vegan/) was used to do Procrustes transformation on the Principal Component coordinates, rotating the PC1 and PC2 obtained for each individual PCA to the configuration of the reference dataset, as previously described [110] (Fig. 1a).

In addition to using smartpca, we confirmed the ancestry of ancient samples with LASER v.2.01 software [126], which was originally designed to account for population structure using low coverage read data. Briefly, we called bases using SAMtools mpileup (-q 30 -Q 20) and used the python script "pileup2seq.py" to convert the pileup format into sequencefile format. We used the Human Genome Diversity Panel (HDGP) dataset [127] provided with this software, already lifted to b37 (http://csg.sph.umich.edu/chaolong/LASER/). We ran LASER with default parameters, which implements Procrustes analysis to project the ancient samples into PCA space (Supplementary Fig. 10).

2.5.2 Projection of Ancient samples

To further resolve genetic affinity of ancient samples to northwestern European populations, we merged the WTCCC1 1958 British Birth Cohort SNP genotype data [111] with data from Dutch [113] and Irish [112] studies. Calls from ancient samples were merged simultaneously with this dataset and filtered by genotyping rate and MAF as above. Principal Component Analysis was computed with smartpca by projecting the ancient individuals onto a PCA constructed with present-day samples. We then perform a Mann-Whitney to investigate if PC1 coordinates differ significantly between groups.

2.6 – Model-based clustering analysis

2.6.1 ADMIXTURE software

PLINK v.1.9 was used to filter the Hellenthal et al. [108] dataset for LD (r2 > 0.1), leaving 67.263 SNPs for analysis, on which we ran ADMIXTURE v.1.23 [128], for clusters (K) ranging from 2 to 10, using time as

seed. The lowest CV error obtained was for K=3. Individual Admixture plots are shown in (Supplementary Fig. 11).

2.6.2 NGSadmix software

Genotype Likelihoods (GLs) in BEAGLE format were obtained from aligned reads using ANGSD v.0.592 [119] a method that takes genotype uncertainties into consideration. Regarding the present-day genotype data, we obtained GLs for SNPs belonging to the 780 individuals using PLINK/SEQ v.0.10 (https://atgu.mgh.harvard.edu/plinkseq/). GLs shared by all ancient and modern samples were then used by NGSadmix v.32 [129] to estimate admixture proportions. Results were plotted with distruct v.1.1 [130] (Fig. 1b).

2.7 Identity-by-state

Merged datasets including each ancient sample and the subset of present-day individuals described above was used for determining Identity-by-state (IBS) relationships between individuals using PLINK v.1.9 [122] (--distance square ibs). After selecting IBS proportions between all modern samples and each ancient, the median IBS for each population was calculated and plotted individually in maps (Supplementary Fig. 12). We then selected all Driffield Terrace individuals, except for 3DRIF-26 (because of the affinity of this sample with Middle Eastern individuals), and ranked their median IBS score in relation to modern populations. The ranks obtained were combined by calculating their product and plotted in Fig. 2. We then calculated Spearman rank correlations between ancient samples based on IBS ranks using the R package "psych" [131] (Supplementary Table 12) and plotted a correlogram on Supplementary Fig. 13 with "corrgram" (http://CRAN.R-project.org/package=corrgram). All 6 Roman York samples with northwestern European affinity (i.e. excluding 3DRIF-26) are very highly correlated in terms of IBS ($r=0.982$, $p<0.01$). In addition, we also tested the IBS approach for sensitivity and specificity (Supplementary Fig. 15).

2.8 Y-chromosome and mtDNA haplogroup identification

2.8.1 Y-chromosome lineage determination

Y-chromosome lineages of ancient male samples were identified using clean_tree software [132] (Table 1). This software calls genotypes using SAMtools mpileup at informative SNPs and determines whether the ancient samples present a derived or ancestral allele. The 539 SNPs used for haplogroup determination were the ones provided by clean_tree which are based on ISOGG 2013 (International Society of Genetic Geneology; http://www.isogg.org/). In addition, we added M269 which was absent in the SNP list used as well as important SNPs for downstream subhaplogroup identification (markers S28 and L11). Due to the low coverage of our samples, 1X was the minimum coverage accepted for polymorphisms to be considered and therefore caution is necessary when interpreting these results, since it is likely that some of the alleles identified result of aDNA damage. Derived alleles for each sample are shown in Supplementary Table 10.

Together these results show marked differences at the level of Y-chromosome haplogroups between samples from the different time periods here analysed. It has recently been suggested that R1b lineages have spread from the East to the West of Europe during the Late Neolithic/Bronze Age [133]. In R1b-derived European Y-chromosomes, L52 lineages are present in more than 90% of Europeans [134,135], and are characterized by a frequency gradient towards the West of Europe. There is a resemblance between R1b-M269 and L52, except that the latter is seen at much lower frequencies in the East of Europe [136]. This trend is also observed at the level of the British Isles, where L52 reaches its highest frequencies in Wales, Ireland and Scotland [136]. All Roman York samples were assigned to L52/L11, either by direct observation of the derived alleles, or by belonging to sub-lineages that imply membership to this branch. Deeper phylogenetic resolution was achieved for some of the samples: 3DRIF-16 and 6DRIF-3 are derived for S21/M405, which can be detected in ~60% of M269+ Y-chromosomes in Central Europe, 6DRIF-21 belongs to DF63, a sub-lineage of S145/L21, more commonly found in the British Isles, in particular Ireland and Wales [136], and 6DRIF-22 was identified as belonging to haplogroup S28, present in modern-day Switzerland, Italy and France, but also reaches over 15% in certain English and German regions [137]. Sample 3DRIF-26 is clearly an exception, both in terms of autosomal variation as in the Y-chromosome lineage it presents (J2), common in the Middle East, Caucasus, Balkans and Italy [138] and attributed to neolithic demic migrations [138] or to seafaring Phoenicians [139]. The Anglo-Saxon sample (NO3423) was assigned to I1 lineage, characterized by higher frequencies Nordic countries [140] and in a british context it is more common in East/Central England with a decrease in frequency towards the West and this distribution has been interpreted as Anglo-Saxon mass migrations into Britain [141].

2.8.2 mtDNA haplogroup identification

Next-Generation Sequencing reads were aligned to the revised Cambridge Reference Sequence (rCRS; NC_012920.1)[117], using BWA filtering for base ($q \geq 30$) and mapping ($q \geq 30$) quality and duplicate reads were removed. We then used SAMtools [107] to obtain the mtDNA consensus for each ancient sample, which was then uploaded to HaploFind (https://haplofind.unibo.it; [118]), where mitochondrial haplogroup defining positions were obtained (Table 1 and Supplementary Table 9). Additionally, we also uploaded alignments in the bam format to mtDNA-Server (http://mtdna-server.uibk.ac.at/; beta version), where heteroplasmy and mtDNA coverage was estimated.

2.9 - ChromoPainter/fineSTRUCTURE analysis

We randomly selected 100 individuals from each region of the WTCCC1 dataset, excluding SNPs with missing genotypes, which resulted in a total of 431,366 variants and 1,000 samples. We split the data by chromosome using PLINK and phased genotypes using SHAPEIT v2.r778[142] . We ran the ChromoPainter pipeline, linked mode, [143] with default parameters as implemented by fineSTRUCTURE v.2. First, "hap" files were converted to ChromoPainter format using "impute2chromopainter.pl", after which we also created a recombination map with "makeuniformrecfile.pl" (downloaded at http://www.paintmychromosomes.com/). The first step consists of "mu" and "Ne" estimation, followed by estimating the "c" parameter [143]. We have ran these computational tasks in HPC mode, by using fineSTRUCTURE to write the necessary commands to run the analysis and combine the output before starting the subsequent step. "Chromocombine" was then used to create a genome wide ChromoPainter

output for all individuals. For the fineSTRUCTURE analysis, the following settings used were: 3,000,000 burn in iterations, 1,000,000 sample iterations for the MCMC and 10,000,000 tree comparisons. We then used GATK to call genotypes in the ancient samples of the present study and converted these to PLINK format. Each ancient individual was merged separately with the WTCCC1 dataset, excluding missing genotypes, followed by estimation of the proportion of IBS between these and the inferred fineSTRUCTURE population clusters (Fig.4, Supplementary Figs. 16, 17). Regression analysis on median values of IBS was performed using the "visreg" package [144] of the R programming language [145].

2.10 - Imputation of phenotypic loci in ancient genomes

The average coverage of genomes is ~1x, therefore we only have access to haploid calls for the majority of SNPs. In order to investigate loci associated with selective sweeps, we took a similar approach as the one described in [99], where alleles observed in the 1000 Genomes Project [146] were called with GATK. Next, we extracted genotype likelihoods and converted these into BEAGLE format. BEAGLE v.3.3.2 [147] was used to phase and subsequently impute genotypes at SNP positions described in the HIrisplex system [148], loci associated with blood groups [149,150], lactase persistence [151,152] and pigmentation phenotypes [153,154]. Only posterior genotype probabilities greater or equal than 0.85 were kept [99] (Supplementary Table 14). We used the HIrisPlex Eye and Hair Colour DNA Phenotyping Webtool [148]; http://www.erasmusmc.nl/47743/3604975/HIris) to predict the ancient samples phenotype as shown in SI Supplementary Table 15. Although phenotype determination based on imputed genotypes has been validated in a previous study [99], it should be regarded as tentative in the present work due to low genomic coverage.

2.10.1 Pigmentation

All samples are homozygous for the selected allele of SLC24A5 SNP (rs1426654), except for the Anglo-Saxon, which is the only heterozygous. For SLC45A2, all samples are homozygous for the selected allele, except for 6DRIF-21 and 6DRIF-22, which are heterozygous. Regarding TYRP1, the following samples are homozygous selected allele: the Iron Age sample and 3DRIF-26. All other samples are heterozygous, except for 6DRIF-18, which is homozygous for the reference allele (Supplementary Table 16).

2.10.2 Lactase persistence

The selected allele of at the LCT locus rs4988235 was identified by imputation in the heterozygous form in the Iron Age individual, and homozygous in sample 6DRIF-3. It was absent in all other samples (Supplementary Table 16).

2.10.3 The Hirisplex system

The majority of the Roman York samples (6 of 7) were determined to have black/brown or blonde/brown hair and brown eyes, with the only exception being 6DRIF-18, who was predicted to have blonde hair and blue eyes, phenotype also shared with the Anglo-Saxon. *The SNP loci rs12913832 at the HERC2/OCA2 locus which determines blue eyes in Europeans, has recently been found to be (weakly) negatively selected and subject of local adaptation [155]. The Anglo-Saxon individual presumably had its origins in northern regions of Europe, where blue eye colour and blonde hair reach very high frequencies in

present-day populations, and therefore it is plausible that the phenotype determined for this individual is accurate. It's worth noticing that the Iron age (M1489) individual of the present study was also determined to have blonde/brown hair, as the majority of the Driffield Terrace samples. Curiously, an Iron Age individual [99] also had the same characteristic.

2.10.4 Blood group determination

By inferring the genotype at 3 loci involved in blood type (rs8176719 [149,150], rs8176746 and rs8176747 [150]), we determined that the majority of Driffield Terrace samples belong to blood group O (6 of 7), with the only exception being 6DRIF-22, which is likely to be type A (Supplementary Table 17). Interestingly, the Iron Age sample is also blood type O. The Anglo-Saxon (NO3423) is likely to be type B and possibly type A.

2.10.5 Frequency maps

Interpolated frequency maps of blood group [156] and Y-chromosome frequency data[141,157,158] were produced using version 10.1 of ArcMap software from the ArcGis suite (Environmental Systems Research Institute) using the default settings of the geospatial analysis plugin.

References

1. Chenery, C., Müldner, G., Evans, J., Eckardt, H. & Lewis, M. Strontium and stable isotope evidence for diet and mobility in Roman Gloucester, UK. *J. Archaeol. Sci.* **37,** 150–163 (2010).
2. Chenery, C., Eckardt, H. & Müldner, G. Cosmopolitan Catterick? Isotopic evidence for population mobility on Rome's northern frontier. *J. Archaeol. Sci.* (2011). at <http://www.sciencedirect.com/science/article/pii/S0305440311000574>
3. Eckardt, H. *et al.* Oxygen and strontium isotope evidence for mobility in Roman Winchester. *J. Archaeol. Sci.* **36,** 2816–2825 (2009).
4. Evans, J. A., Chenery, C. A. & Montgomery, J. A summary of strontium and oxygen isotope variation in archaeological human tooth enamel excavated from Britain. *J. Anal. At. Spectrom.* **27,** 754–764 (2012).
5. Montgomery, J., Evans, J. A., Chenery, S. R., Pashley, V. & Killgrove, K. 'Gleaming, white and deadly': using lead to track human exposure and geographic origins in the Roman period in Britain. *Journal of Roman archaeology; supplementary series.* 199–226 (2010).
6. Müldner, G., Chenery, C. & Eckardt, H. The 'Headless Romans': multi-isotope investigations of an unusual burial ground from Roman Britain. *J. Archaeol. Sci.* **38,** 280–290 (2011).
7. Müldner, G. & Richards, M. P. Stable isotope evidence for 1500 years of human diet at the city of York, UK. *Am. J. Phys. Anthropol.* **133,** 682–697 (2007).
8. Müldner, G. Stable isotopes and diet: their contribution to Romano-British research. *Antiquity* (2013). at <http://www.antiquity.ac.uk/projgall/muldner335/downloads/muldner335_supplement.pdf>
9. Dupras, L. T. Dining in the Dakhleh Oasis, Egypt: Determination of diet using documents and stable isotope analysis. (1999). at <https://macsphere.mcmaster.ca/handle/11375/6565>
10. Iacumin, P., Bocherens, H., Mariotti, A. & Longinelli, A. Oxygen isotope analyses of co-existing carbonate and phosphate in biogenic apatite: a way to monitor diagenetic alteration of bone phosphate? *Earth Planet. Sci. Lett.* **142,** 1–6 (1996).
11. Thompson, A. H., Richards, M. P., Shortland, A. & Zakrzewski, S. R. Isotopic palaeodiet studies of ancient

Egyptian fauna and humans. *J. Archaeol. Sci.* **32,** 451–463 (2005).

12. Thompson, A. H., Chaix, L. & Richards, M. P. Stable isotopes and diet at Ancient Kerma, Upper Nubia (Sudan). *J. Archaeol. Sci.* **35,** 376–387 (2008).
13. White, C. D. & Schwarcz, H. P. Temporal trends in stable isotopes for Nubian mummy tissues. *Am. J. Phys. Anthropol.* **93,** 165–187 (1994).
14. Keenleyside, A., Schwarcz, H., Stirling, L. & Ben Lazreg, N. Stable isotopic evidence for diet in a Roman and Late Roman population from Leptiminus, Tunisia. *J. Archaeol. Sci.* **36,** 51–63 (2009).
15. Sandias, M. The reconstruction of diet and environment in ancient Jordan by carbon and nitrogen stable isotope analysis of human and animal remains. *Water, life and civilisation. Climate, environment and society in the Jordan Valley* 337–346 (2011).
16. Bashaireh, K. Al-, Shorman, A. Al-, Rose, J., Jull, A. J. T. & Hodgins, G. Paleodiet Reconstruction of Human Remains from the Archaeological Site of Natfieh, Northern Jordan. *Radiocarbon* **52,** 645–652 (2010).
17. Sołtysiak, A. & Schutkowski, H. Continuity and change in subsistence at Tell Barri, NE Syria. *Journal of Archaeological Science: Reports* **2,** 176–185 (2015).
18. Fuller, B. T. *et al.* Isotopic reconstruction of human diet and animal husbandry practices during the Classical-Hellenistic, imperial, and Byzantine periods at Sagalassos, Turkey. *Am. J. Phys. Anthropol.* **149,** 157–171 (2012).
19. Sandias, M. & Müldner, G. Diet and herding strategies in a changing environment: Stable isotope analysis of Bronze Age and Late Antique skeletal remains from Ya'amūn, Jordan. *J. Archaeol. Sci.* **63,** 24–32 (2015).
20. Bourbou, C., Fuller, B. T., Garvie-Lok, S. J. & Richards, M. P. Reconstructing the diets of Greek Byzantine populations (6th–15th centuries AD) using carbon and nitrogen stable isotope ratios. *Am. J. Phys. Anthropol.* **146,** 569–581 (2011).
21. Killgrove, K. & Tykot, R. H. Food for Rome: A stable isotope investigation of diet in the Imperial period (1st–3rd centuries AD). *Journal of Anthropological Archaeology* **32,** 28–38 (2013).
22. Prowse, T., Schwarcz, H. P., Saunders, S., Macchiarelli, R. & Bondioli, L. Isotopic paleodiet studies of skeletons from the Imperial Roman-age cemetery of Isola Sacra, Rome, Italy. *J. Archaeol. Sci.* **31,** 259–272 (2004).

23. Craig, O. E. *et al.* Stable isotopic evidence for diet at the Imperial Roman coastal site of Velia (1st and 2nd centuries AD) in Southern Italy. *Am. J. Phys. Anthropol.* **139,** 572–583 (2009).

24. Crowe, F. *et al.* Water-related occupations and diet in two Roman coastal communities (Italy, first to third century AD): correlation between stable carbon and nitrogen isotope values and auricular exostosis prevalence. *Am. J. Phys. Anthropol.* **142,** 355–366 (2010).

25. Fuller, B. T., Márquez-Grant, N. & Richards, M. P. Investigation of diachronic dietary patterns on the islands of Ibiza and formentera, Spain: Evidence from carbon and nitrogen stable isotope ratio analysis. *Am. J. Phys. Anthropol.* **143,** 512–522 (2010).

26. Eriksson, G. *et al.* Same island, different diet: Cultural evolution of food practice on Öland, Sweden, from the Mesolithic to the Roman Period. *Journal of Anthropological Archaeology* **27,** 520–543 (2008).

27. Lightfoot, E., Slaus, M. & O'Connell, T. C. Changing cultures, changing cuisines: Cultural transitions and dietary change in Iron Age, Roman, and Early Medieval Croatia. *Am. J. Phys. Anthropol.* **148,** 543–556 (2012).

28. Jørkov, M. L. S., Jørgensen, L. & Lynnerup, N. Uniform diet in a diverse society. Revealing new dietary evidence of the Danish Roman Iron Age based on stable isotope analysis. *Am. J. Phys. Anthropol.* **143,** 523–533 (2010).

29. Reitsema, L. J. & Kozłowski, T. Diet and society in Poland before the state: stable isotope evidence from a Wielbark population (2nd c. AD). *Anthropological Review* **76,** 1–22

30. Hakenbeck, S., McManus, E., Geisler, H., Grupe, G. & O'Connell, T. Diet and mobility in Early Medieval Bavaria: a study of carbon and nitrogen stable isotopes. *Am. J. Phys. Anthropol.* **143,** 235–249 (2010).

31. Ottaway, P. *Roman York*. (Tempus Pub Limited, 2004).

32. Hunter-Mann, K. *6 Driffield Terrace, York: assessment report on an archaeological excavation*. (York Archaeology Trust, 2005).

33. Ottaway, P. *1–3 Driffield Terrace, York. Assessment Report on an Archaeological Excavation, 2005*. (York Archaeological Trust for Excavation and Research, 2005).

34. Caffell, A. & Holst, M. *Osteological Analysis, 3 and 6 Driffield Terrace, York, North Yorkshire*. (York Osteoarchaeology, 2012).

35. Roberts, C. A. & Manchester, K. Archaeology of disease. (DU, 2005).

36. Kanz, F. & Grossschmidt, K. Head injuries of Roman gladiators. *Forensic Sci. Int.* **160,** 207–216 (2006).

37. Galloway, A. Fracture patterns and skeletal morphology: the upper extremity. *Broken bones: anthropological analysis of blunt force trauma. Springfield, IL: CC Thomas. p* 113–159 (1999).

38. Cannon, S. R., Dowd, G. S., Williams, D. H. & Scott, J. M. A long-term study following Bennett's fracture. *J. Hand Surg. Br.* **11,** 426–431 (1986).

39. Dandy, D. J. & Edwards, D. J. *Essential orthopaedics and trauma*. (Elsevier Health Sciences, 2009).

40. Blondiaux, J. *et al.* Bilateral fractures of the scapula: Possible archeological examples of beatings from Europe, Africa and America. *International Journal of Paleopathology* **2,** 223–230 (2012).

41. Tucker, K. *The inhumations from 1-3 and 6 Driffield Terrace', Unpublished Preliminary Assessment Report*. (York Archaeological Trust , 2006).

42. Ortner, D. J. *Identification of Pathological Conditions in Human Skeletal Remains*. (Elsevier Science, 2003).

43. Walker, P. L., Bathurst, R. R., Richman, R., Gjerdrum, T. & Andrushko, V. A. The causes of porotic hyperostosis and cribra orbitalia: A reappraisal of the iron-deficiency-anemia hypothesis. *Am. J. Phys. Anthropol.* **139,** 109–125 (2009).

44. Roberts, C. A. & Cox, M. *Health and disease in Britain: from prehistory to the present day*. (Sutton publishing, 2003).

45. Holst, M. *Osteological Analysis, Mill Mount, York*. (York Osteoarchaeology, 2006).

46. Fenton-Thomas, C. *Where Sky and Yorkshire and Water Meet: The Story of the Melton Landscape from Prehistory to the Present: Archaeological Excavations on the A63 Grade Separated Junction at Melton, East Yorkshire*. (On-Site Archaeology, 2011).

47. Sherlock, S. J. & Welch, M. G. *An Anglo-Saxon Cemetery at Norton, Cleveland*. **82,** (Council for British Archaeology York, 1992).

48. Johnson, P. *Cemetery excavations at Bishopsmill School, Norton: Post-excavation assessment and analysis*. (Teeside Archaeology, 2005).

49. Montgomery, J., Knüsel, C. J. & Tucker, K. in *The bioarchaeology of the human head : decapitation, decoration and deformation.* (ed. Bonogofsky, M.) 141–178 (University Press of Florida, 2011).

50. Müldner, G., Chenery, C. & Eckardt, H. The 'Headless Romans': multi-isotope investigations of an unusual

burial ground from Roman Britain. *J. Archaeol. Sci.* **38,** 280–290 (2011).

51. Montgomery, J. Lead and strontium isotape compositions of human dental tissues as an indicator of ancient exposure and population dynamics. (The University of Bradford, 2002).
52. Chenery, C. A., Pashley, V., Lamb, A. L., Sloane, H. J. & Evans, J. A. The oxygen isotope relationship between the phosphate and structural carbonate fractions of human bioapatite. *Rapid Commun. Mass Spectrom.* **26,** 309–319 (2012).
53. Daux, V. *et al.* Oxygen isotope fractionation between human phosphate and water revisited. *J. Hum. Evol.* **55,** 1138–1147 (2008).
54. Longin, R. New method of collagen extraction for radiocarbon dating. *Nature* **230,** 241–242 (1971).
55. Collins, M. J. & Galley, P. Towards an optimal method of archaeological collagen extraction: the influence of pH and grinding. *Anc. Biomol.* **2,** 209–223 (1998).
56. DeNiro, M. J. Postmortem preservation and alteration of in vivo bone collagen isotope ratios in relation to palaeodietary reconstruction. *Nature* **317,** 806–809 (1985).
57. Van Klinken, G. Bone Collagen Quality Indicators for Palaeodietary and Radiocarbon Measurements. *J. Archaeol. Sci.* **26,** 687–695 (1999).
58. Evans, J. A., Montgomery, J., Wildman, G. & Boulton, N. Spatial variations in biosphere 87Sr/86Sr in Britain. *J. Geol. Soc. London* **167,** 1–4 (2010).
59. Burke, W. H. *et al.* Variation of seawater 87Sr/86Sr throughout Phanerozoic time. *Geology* **10,** 516–519 (1982).
60. Warham, J. O. Mapping biosphere strontium isotope ratios across major lithological boundaries. A systematic investigation of the major influences on geographic variation in the 87Sr/86Sr composition of bioavailable strontium above the Cretaceous and Jurassic rocks of England. (University of Bradford, 2013).
61. Montgomery, J., Evans, J. A. & Cooper, R. E. Resolving archaeological populations with Sr-isotope mixing models. *Appl. Geochem.* **22,** 1502–1514 (2007).
62. Jay, M., Montgomery, J., Nehlich, O., Towers, J. & Evans, J. British Iron Age chariot burials of the Arras culture: a multi-isotope approach to investigating mobility levels and subsistence practices. *World Archaeol.* **45,** 473–491 (2013).

63. Dansgaard, W. Stable isotopes in precipitation. *Tell'Us* **16,** 436–468 (1964).

64. Darling, G., Bath, A. H. & Talbot, J. C. The O and H stable isotope composition of freshwaters in the British Isles. 2, surface waters and groundwater. *Hydrol. Earth Syst. Sci.* **7,** 183–195 (2003).

65. Brettell, R., Montgomery, J. & Evans, J. Brewing and stewing: the effect of culturally mediated behaviour on the oxygen isotope composition of ingested fluids and the implications for human provenance studies. *J. Anal. At. Spectrom.* **27,** 778–785 (2012).

66. Lamb, A. L., Evans, J. E., Buckley, R. & Appleby, J. Multi-isotope analysis demonstrates significant lifestyle changes in King Richard III. *J. Archaeol. Sci.* **50,** 559–565 (2014).

67. Montgomery, J., Evans, J. A., Chenery, C. A. & Müldner, G. Stable isotope analysis of bone. *Wasperton: A Roman, British and Anglo-Saxon Community in Central England. Boydell & Brewer, Woodbridge* 48–49 (2009).

68. Hemer, K., Evans, J., Chenery, C. & Lamb, A. Evidence of early medieval trade and migration between Wales and the Mediterranean Sea region. *J. Archaeol. Sci.* **40,** 2352–2359 (2013).

69. Montgomery, J. *et al.* Finding Vikings with Isotope Analysis: The View from Wet and Windy Islands. *Journal of the North Atlantic* **7,** 54–70 (2014).

70. Montgomery, J., Evans, J. A., Chenery, S. R., Pashley, V. & Killgrove, K. 'Gleaming, white and deadly': using lead to track human exposure and geographic origins in the Roman period in Britain. *Journal of Roman archaeology; supplementary series.* 199–226 (2010).

71. Hillson, S. *Dental anthropology*. (Cambridge University Press, 1996).

72. Richards, M. P., Hedges, R., Molleson, T. I. & Vogel, J. C. Stable isotope analysis reveals variations in human diet at the Poundbury Camp cemetery site. *J. Archaeol. Sci.* **25,** 1247–1252 (1998).

73. Cummings, C. Food and society in late Roman Britain: determining dietary patterns using stable isotope analysis. (University of Oxford, 2008).

74. Lightfoot, E. *et al.* AN INVESTIGATION INTO DIET AT THE SITE OF YARNTON, OXFORDSHIRE, USING STABLE CARBON AND NITROGEN ISOTOPES. *Oxford Journal of Archaeology* **28,** 301–322 (2009).

75. Redfern, R. C., Hamlin, C. & Athfield, N. B. Temporal changes in diet: a stable isotope analysis of late Iron

Age and Roman Dorset, Britain. *J. Archaeol. Sci.* **37,** 1149–1160 (2010).

76. Pollard, A. M. *et al.* 'These boots were made for walking': The isotopic analysis of a C4 Roman inhumation from Gravesend, Kent, UK. *Am. J. Phys. Anthropol.* **146,** 446–456 (2011).
77. Cheung, C., Schroeder, H. & Hedges, R. E. M. Diet, social differentiation and cultural change in Roman Britain: new isotopic evidence from Gloucestershire. *Archaeol. Anthropol. Sci.* **4,** 61–73 (2011).
78. Sealy, J., Armstrong, R. & Schrire, C. Beyond lifetime averages: tracing life histories through isotopic analysis of different calcified tissues from archaeological human skeletons. *Antiquity* **69,** 290–300 (1995).
79. Heaton, T. H. E. The 15N/14N ratios of plants in South Africa and Namibia: relationship to climate and coastal/saline environments. *Oecologia* **74,** 236–246 (1987).
80. Schwarcz, H. P., Dupras, T. L. & Fairgrieve, S. I. 15 N enrichment in the Sahara: in search of a global relationship. *J. Archaeol. Sci.* **26,** 629–636 (1999).
81. Hartman, G. & Danin, A. Isotopic values of plants in relation to water availability in the Eastern Mediterranean region. *Oecologia* **162,** 837–852 (2010).
82. Földvary, G. Z. *Geology of the Carpathian region*. (World Scientific, 1988).
83. Hoffs, J. & Wedepohl, K. H. Strontium isotope studies on young volcanic rocks from Germany and Italy. *Contrib. Mineral. Petrol.* **19,** 328–338 (1968).
84. Asch, K. IGME 5000 Geological Map of Europe and Adjacent Areas. *BGR, Hanover* (2005).
85. Derry, D. R. *A Concise World Atlas of Geology and Mineral Deposits*. (1982).
86. Hartman, G. & Richards, M. Mapping and defining sources of variability in bioavailable strontium isotope ratios in the Eastern Mediterranean. *Geochim. Cosmochim. Acta* **126,** 250–264 (2014).
87. Farber, E. *et al.* The origin and mechanisms of salinization of the lower Jordan river 1. *Geochim. Cosmochim. Acta* **68,** 1989–2006 (2004).
88. Bowen, G. J. & Revenaugh, J. Interpolating the isotopic composition of modern meteoric precipitation. *Water Resour. Res.* **39,** 1299 (2003).
89. Büntgen, U. *et al.* 2500 years of European climate variability and human susceptibility. *Science* **331,** 578–582 (2011).
90. McCormick, M. *et al.* Climate Change during and after the Roman Empire: Reconstructing the Past from

Scientific and Historical Evidence. *J. Interdiscip. Hist.* **43,** 169–220 (2012).

91. Mitchell, P. D. & Millard, A. R. Migration to the medieval Middle East with the crusades. *Am. J. Phys. Anthropol.* **140,** 518–525 (2009).

92. Gat, J. R. & Dansgaard, W. Stable isotope survey of the fresh water occurrences in Israel and the northern Jordan Rift Valley. *J. Hydrol.* **16,** 177–211 (1972).

93. Perry, M. A., Coleman, D. S., Dettman, D. L. & Shiyab, A. H. al-. An isotopic perspective on the transport of Byzantine mining camp laborers into southwestern Jordan. *Am. J. Phys. Anthropol.* **140,** 429–441 (2009).

94. Lykoudis, S. P. & Argiriou, A. A. Gridded data set of the stable isotopic composition of precipitation over the eastern and central Mediterranean. *J. Geophys. Res.* **112,** D18107 (2007).

95. Touzeau, A. *et al.* Diet of ancient Egyptians inferred from stable isotope systematics. *J. Archaeol. Sci.* **46,** 114–124 (2014).

96. Buzon, M. R., Simonetti, A. & Creaser, R. A. Migration in the Nile Valley during the New Kingdom period: a preliminary strontium isotope study. *J. Archaeol. Sci.* **34,** 1391–1401 (2007).

97. Chenery, C., Eckardt, H. & Müldner, G. Cosmopolitan Catterick? Isotopic evidence for population mobility on Rome's Northern frontier. *J. Archaeol. Sci.* **38,** 1525–1536 (2011).

98. Schwarcz, H. & Schoeninger, M. Dupras TL & Fairgrieve SI (1999). 15N enrichment in the Sahara: in search of a global relationship. *J. Archaeol. Sci.* **26,** 629–636

99. Gamba, C. *et al.* Genome flux and stasis in a five millennium transect of European prehistory. *Nat. Commun.* **5,** 5257 (2014).

100. Yang, D. Y., Eng, B., Waye, J. S., Dudar, J. C. & Saunders, S. R. Technical note: improved DNA extraction from ancient bones using silica-based spin columns. *Am. J. Phys. Anthropol.* **105,** 539–543 (1998).

101. MacHugh, D. E., Edwards, C. J., Bailey, J. F., Bancroft, D. R. & Bradley, D. G. The Extraction and Analysis of Ancient DNA From Bone and Teeth: a Survey of Current Methodologies. *Anc. Biomol.* **3,** 81 (2000).

102. Meyer, M. & Kircher, M. Illumina sequencing library preparation for highly multiplexed target capture and sequencing. *Cold Spring Harb. Protoc.* **2010,** db.prot5448 (2010).

103. Martiniano, R. *et al.* Genetic evidence of African slavery at the beginning of the trans-Atlantic slave trade. *Sci. Rep.* **4,** 5994 (2014).

104. Martin, M. Cutadapt removes adapter sequences from high-throughput sequencing reads. *EMBnet.journal* **17,** 10–12 (2011).

105. Li, H. & Durbin, R. Fast and accurate short read alignment with Burrows-Wheeler transform. *Bioinformatics* **25,** 1754–1760 (2009).

106. Schubert, M. *et al.* Improving ancient DNA read mapping against modern reference genomes. *BMC Genomics* **13,** 178 (2012).

107. Li, H. *et al.* The Sequence Alignment / Map (SAM) Format and SAMtools 1000 Genome Project Data Processing Subgroup. *Bioinformatics* **25,** 2078–2079 (2009).

108. Hellenthal, G. *et al.* A genetic atlas of human admixture history. *Science* **343,** 747–751 (2014).

109. Purcell, S. *et al.* PLINK: a tool set for whole-genome association and population-based linkage analyses. *Am. J. Hum. Genet.* **81,** 559–575 (2007).

110. Skoglund, P. *et al.* Origins and Genetic Legacy of Neolithic Farmers and Hunter-Gatherers in Europe. *Science* **336,** 466–469 (2012).

111. Wellcome Trust Case Control Consortium. Genome-wide association study of 14,000 cases of seven common diseases and 3,000 shared controls. *Nature* **447,** 661–678 (2007).

112. McLaughlin, R. L. *et al.* A second-generation Irish genome-wide association study for amyotrophic lateral sclerosis. *Neurobiol. Aging* **36,** 1221.e7–1221.e13 (2015).

113. Van Es, M. A. *et al.* Genome-wide association study identifies 19p13.3 (UNC13A) and 9p21.2 as susceptibility loci for sporadic amyotrophic lateral sclerosis. *Nat. Genet.* **41,** 1083–1087 (2009).

114. Jónsson, H., Ginolhac, A., Schubert, M., Johnson, P. L. F. & Orlando, L. mapDamage2.0: fast approximate Bayesian estimates of ancient DNA damage parameters. *Bioinformatics* **29,** 1682–1684 (2013).

115. McKenna, A. *et al.* The Genome Analysis Toolkit: a MapReduce framework for analyzing next-generation DNA sequencing data. *Genome Res.* **20,** 1297–1303 (2010).

116. Skoglund, P., Storå, J., Götherström, A. & Jakobsson, M. Accurate sex identification of ancient human remains using DNA shotgun sequencing. *J. Archaeol. Sci.* **40,** 4477–4482 (2013).

117. Andrews, R. M. *et al.* Reanalysis and revision of the Cambridge reference sequence for human mitochondrial DNA. *Nat. Genet.* **23,** 147 (1999).

118. Van Oven, M. & Kayser, M. Updated comprehensive phylogenetic tree of global human mitochondrial DNA variation. *Hum. Mutat.* **30,** E386–94 (2009).

119. Korneliussen, T., Albrechtsen, A. & Nielsen, R. ANGSD: Analysis of Next Generation Sequencing Data. *BMC Bioinformatics* **15,** 356 (2014).

120. Rasmussen, M. *et al.* An Aboriginal Australian Genome Reveals Separate Human Dispersals into Asia. *Science* **334 ,** 94–98 (2011).

121. Skoglund, P. *et al.* Separating endogenous ancient DNA from modern day contamination in a Siberian Neandertal. *Proc. Natl. Acad. Sci. U. S. A.* **111,** 2229–2234 (2014).

122. Chang, C. C. *et al.* Second-generation PLINK: rising to the challenge of larger and richer datasets. *arXiv [q-bio.GN]* (2014).

123. Patterson, N., Price, A. L. & Reich, D. Population structure and eigenanalysis. *PLoS Genet.* **2,** 2074–2093 (2006).

124. Price, A. L. *et al.* Principal components analysis corrects for stratification in genome-wide association studies. *Nat. Genet.* **38,** 904–909 (2006).

125. R Development Core Team, R. *R: A Language and Environment for Statistical Computing*. (2011).

126. Wang, C. *et al.* Ancestry estimation and control of population stratification for sequence-based association studies. *Nat. Genet.* **46,** 409–415 (2014).

127. Li, J. Z. *et al.* Worldwide human relationships inferred from genome-wide patterns of variation. *Science* **319,** 1100–1104 (2008).

128. Alexander, D. H., Novembre, J. & Lange, K. Fast model-based estimation of ancestry in unrelated individuals. *Genome Res.* **19,** 1655–1664 (2009).

129. Skotte, L., Korneliussen, T. S. & Albrechtsen, A. Estimating individual admixture proportions from next generation sequencing data. *Genetics* **195,** 693–702 (2013).

130. Rosenberg, N. A. DISTRUCT: a program for the graphical display of population structure. *Mol. Ecol. Notes* **4,** 137–138 (2004).

131. Revelle, W. psych: Procedures for personality and psychological research. *Northwestern University, Evanston. R package version* **1,** (2014).

132. Ralf, A., van Oven, M., Zhong, K. & Kayser, M. Simultaneous analysis of hundreds of Y-chromosomal SNPs for high-resolution paternal lineage classification using targeted semiconductor sequencing. *Hum. Mutat.* **36,** 151–159 (2015).

133. Haak, W. *et al.* Massive migration from the steppe was a source for Indo-European languages in Europe. *Nature* (2015). doi:10.1038/nature14317

134. Rootsi, S. *et al.* Phylogenetic applications of whole Y-chromosome sequences and the Near Eastern origin of Ashkenazi Levites. *Nat. Commun.* **4,** 2928 (2013).

135. Rocca, R. A. *et al.* Discovery of Western European R1b1a2 Y chromosome variants in 1000 genomes project data: an online community approach. *PLoS One* **7,** e41634 (2012).

136. Busby, G. B. J. *et al.* The peopling of Europe and the cautionary tale of Y chromosome lineage R-M269. *Proc. Biol. Sci.* **279,** 884–892 (2012).

137. Myres, N. M. *et al.* A major Y-chromosome haplogroup R1b Holocene era founder effect in Central and Western Europe. *Eur. J. Hum. Genet.* **19,** 95–101 (2011).

138. Semino, O. *et al.* Origin, diffusion, and differentiation of Y-chromosome haplogroups E and J: inferences on the neolithization of Europe and later migratory events in the Mediterranean area. *Am. J. Hum. Genet.* **74,** 1023–1034 (2004).

139. Zalloua, P. A. *et al.* Identifying genetic traces of historical expansions: Phoenician footprints in the Mediterranean. *Am. J. Hum. Genet.* **83,** 633–642 (2008).

140. Lappalainen, T. *et al.* Migration waves to the Baltic Sea region. *Ann. Hum. Genet.* **72,** 337–348 (2008).

141. Weale, M. E., Weiss, D. A., Jager, R. F., Bradman, N. & Thomas, M. G. Y chromosome evidence for Anglo-Saxon mass migration. *Mol. Biol. Evol.* **19,** 1008–1021 (2002).

142. Delaneau, O., Marchini, J. & Zagury, J.-F. A linear complexity phasing method for thousands of genomes. *Nat. Methods* **9,** 179–181 (2012).

143. Lawson, D. J., Hellenthal, G., Myers, S. & Falush, D. Inference of population structure using dense haplotype data. *PLoS Genet.* **8,** e1002453 (2012).

144. Breheny, P. & Burchett, W. visreg: Visualization of regression models. *R package version* 2–0 (2012).

145. Team, R. C. R: A language and environment for statistical computing. R Foundation for Statistical Computing,

Vienna, Austria, 2012. (2014).

146. 1000 Genomes Project Consortium *et al.* A map of human genome variation from population-scale sequencing. *Nature* **467,** 1061–1073 (2010).

147. Browning, B. L. & Browning, S. R. A fast, powerful method for detecting identity by descent. *Am. J. Hum. Genet.* **88,** 173–182 (2011).

148. Walsh, S. *et al.* The HIrisPlex system for simultaneous prediction of hair and eye colour from DNA. *Forensic Sci. Int. Genet.* **7,** 98–115 (2013).

149. Heit, J. A. *et al.* A genome-wide association study of venous thromboembolism identifies risk variants in chromosomes 1q24.2 and 9q. *J. Thromb. Haemost.* **10,** 1521–1531 (2012).

150. Nakao, M. *et al.* ABO blood group alleles and the risk of pancreatic cancer in a Japanese population. *Cancer Sci.* **102,** 1076–1080 (2011).

151. Bersaglieri, T. *et al.* Genetic signatures of strong recent positive selection at the lactase gene. *Am. J. Hum. Genet.* **74,** 1111–1120 (2004).

152. Enattah, N. S. *et al.* Identification of a variant associated with adult-type hypolactasia. *Nat. Genet.* **30,** 233–237 (2002).

153. Beleza, S. *et al.* Genetic architecture of skin and eye color in an African-European admixed population. *PLoS Genet.* **9,** e1003372 (2013).

154. Canfield, V. A. *et al.* Molecular phylogeography of a human autosomal skin color locus under natural selection. *G3* **3,** 2059–2067 (2013).

155. Mathieson, I. *et al. Eight thousand years of natural selection in Europe*. (2015).

156. Cavalli-Sforza, L. L., Menozzi, P. & Piazza, A. *The history and geography of human genes*. (Princeton university press, 1994).

157. Moore, L. T., Mc Evoy, B., Cape, E., Simms, K. & Bradley, D. G. Report A Y-Chromosome Signature of Hegemony in Gaelic Ireland. *Am. J. Hum. Genet.* **78,** 334–338 (2006).

158. Capelli, C. *et al.* A Y chromosome census of the British Isles. *Curr. Biol.* **13,** 979–984 (2003).
